# The effects of neuromuscular electrical stimulation on hospitalised adults: systematic review and meta-analysis of randomised controlled trials

**DOI:** 10.1093/ageing/afad236

**Published:** 2023-12-28

**Authors:** Helal B Alqurashi, Katie Robinson, Dominic O’Connor, Mathew Piasecki, Adam L Gordon, Tahir Masud, John R F Gladman

**Affiliations:** University of Nottingham, Nottingham, UK; Department of Physical Therapy, Faculty of Applied Medical Science, Taif University, Taif, Saudi Arabia; NIHR Nottingham Biomedical Research Centre (BRC), UK; University of Nottingham, Nottingham, UK; NIHR Nottingham Biomedical Research Centre (BRC), UK; Nottingham University Hospitals NHS Trust, Nottingham, UK; University of Nottingham, Nottingham, UK; University of Nottingham, Nottingham, UK; NIHR Nottingham Biomedical Research Centre (BRC), UK; University of Nottingham, Nottingham, UK; NIHR Nottingham Biomedical Research Centre (BRC), UK; NIHR Applied Research Collaboration (ARC) East Midlands, UK; University Hospitals of Derby and Burton NHS Foundation Trust, Derby, UK; NIHR Nottingham Biomedical Research Centre (BRC), UK; Nottingham University Hospitals NHS Trust, Nottingham, UK; University of Nottingham, Nottingham, UK; NIHR Nottingham Biomedical Research Centre (BRC), UK; Nottingham University Hospitals NHS Trust, Nottingham, UK; NIHR Applied Research Collaboration (ARC) East Midlands, UK

**Keywords:** neuromuscular electrical stimulation, hospital-acquired disability, muscle strength, physical function, systematic review, older people

## Abstract

**Introduction:**

Neuromuscular electrical stimulation (NMES) is a treatment to prevent or reverse acquired disability in hospitalised adults. We conducted a systematic review and meta-analysis of its effectiveness.

**Method:**

We searched MEDLINE, EMBASE, Cumulative Index to Nursing & Allied Health (CINAHL) and the Cochrane library. Inclusion criteria: randomised controlled trials of hospitalised adult patients comparing NMES to control or usual care. The primary outcome was muscle strength. Secondary outcomes were muscle size, function, hospital length of stay, molecular and cellular biomarkers, and adverse effects. We assessed risk of bias using the Cochrane risk-of-bias tool. We used Review Manager (RevMan) software for data extraction, critical appraisal and synthesis. We assessed certainty using the Grading of Recommendations Assessment, Development and Evaluation tool.

**Results:**

A total of 42 papers were included involving 1,452 participants. Most studies had unclear or high risk of bias. NMES had a small effect on muscle strength (moderate certainty) (standardised mean difference (SMD) = 0.33; *P* < 0.00001), a moderate effect on muscle size (moderate certainty) (SMD = 0.66; *P* < 0.005), a small effect on walking performance (moderate certainty) (SMD = 0.48; *P* < 0.0001) and a small effect on functional mobility (low certainty) (SMD = 0.31; *P* < 0.05). There was a small and non-significant effect on health-related quality of life (very low certainty) (SMD = 0.35; *P* > 0.05). In total, 9% of participants reported undesirable experiences. The effects of NMES on length of hospital stay, and molecular and cellular biomarkers were unclear.

**Conclusions:**

NMES is a promising intervention component that might help to reduce or prevent hospital-acquired disability.

## Key Points

- Neuromuscular electrical stimulation is a potential intervention to reduce hospital-acquired disability.
- Neuromuscular electrical stimulation improves muscle strength, size, walking and functioning performance in hospitalised adults.
- Further applied research should optimise the stimulation parameters and evaluate its contribution to rehabilitation programmes.

## Introduction

People admitted to hospital frequently develop hospital-acquired disability [1]. This is partly due to loss of muscle mass and function, in turn due to factors including immobilisation, inflammation and malnutrition [2].

Early rehabilitation using exercise improves outcomes in hospital patients [3–5]. In practice, however, many patients are medically unstable or experience symptoms that render exercise unfeasible [6, 7]. An alternative or additional intervention is neuromuscular electrical stimulation (NMES), in which involuntary muscle contraction occurs from non-invasive, low-frequency current transmitted through electrodes typically placed over thigh and leg muscles. Patients can use NMES in bed or seated, with or without voluntary effort [8, 9].

Previous systematic reviews of NMES have shown inconsistent effects in conditions including elective surgery [10], neurological disorders [11, 12], osteoarthritis [13], chronic obstructive pulmonary disease (COPD) [14, 15], heart failure [16], advanced diseases (chronic respiratory disease, chronic heart failure, cancer or HIV/AIDS) [17], cancer [18] and intensive care unit (ICU) patients [15, 19, 20]. However, no review has focused on the effectiveness of lower limb NMES in hospitalised adults. We report a systematic review and meta-analysis to examine evidence for the effects of NMES in hospitalised adults.

## Methods

The protocol followed PRISMA-P guidelines [21] and was registered at the International Prospective Register of Systematic Reviews (PROSPERO; registration number: CRD42021259763).

## Eligibility criteria

### Inclusion criteria

- Participants: adults (aged ≥18 years) hospitalised with acute medical or acute or elective surgical conditions.
- Intervention: NMES applied to a limb, whether given as a single intervention or in combination with other interventions.
- Control: no, sham treatment or other usual care.
- Outcomes: including one or more of the outcomes of interest.
  - Primary outcomes: muscle strength: chosen as the most immediate and direct benefit of NMES.
  - Secondary outcomes, including the following outcomes and categories:
    ▪ Muscle size: sarcopenia is the combination of reduced muscle strength and mass.
    ▪ Function: to examine whether any benefits of NMES translate into functional gains.
    ▪ Hospital length of stay: economic importance.
    ▪ Molecular and cellular (fibre type composition; inflammatory mediators; muscle protein synthesis and breakdown; bone; lipid and lipoprotein markers): to illuminate the mechanism of action of NMES and identify biomarkers.
    ▪ Adverse effects: the decision to use NMES is a balance between benefits and harms.
- Study design: randomised controlled trials (RCTs) and quasi-randomised controlled trials.

### Exclusion criteria

- Participants: patients selected due to psychiatric, speech, swallowing or facial disorders.
- Intervention: NMES superimposed onto movement or not applied to a limb (e.g. solely applied to treat facial, swallowing or speech problems); electrical stimulation used for its afferent effect such as for pain or spasticity rather than to produce muscular stimulation (transcutaneous elections stimulation); or pulsed electrical stimulation to augment normal movement such as functional electrical stimulation.
- Control: where any control conditions, treatments or interventions other than NMES were different from the intervention group, for example if NMES was given with an exercise programme that was not given to the control group, or where the control group had an exercise programme not given to the NMES group; where the control comparison only a difference between NMES parameters.
- Reporting: not published in English, for researcher resource reasons.

### Information sources

MEDLINE, EMBASE, Cumulative Index to Nursing & Allied Health (CINAHL) and Cochrane library electronic databases were searched from inception to 18 February 2023. Trial registers were not searched for unpublished studies, which may be less reliable than studies that have been through peer review.

### Search strategy

Keywords used to perform the search were adults AND hospitalised AND critically ill AND neuromuscular electrical stimulation (supplementary file). Reference lists of selected studies were searched for additional studies.

### Selection process

Two reviewers independently screened titles and abstracts against eligibility criteria, using Rayyan software. From those included at this stage, two reviewers independently examined full-text articles against eligibility criteria. Any disagreements were resolved by discussion or by a third reviewer.

### Data extraction

Two reviewers independently performed data extraction for all included studies using a standardised data extraction table. Information extracted included study information (first author, publication year, country, design, study period and setting), participant characteristics (conditions, total sample size, gender, age), intervention group (sample size, gender, age, detailed protocol parameters, additional intervention), control group (sample size, gender, age, intervention type), outcome measures, follow-up, results, dropout and limitations.

### Risk-of-bias assessment

Two reviewers independently performed risk-of-bias assessments (RoB) using a third reviewer to resolve disagreements. The Cochrane Collaboration RoB assessment tool for randomised trials [22] was used, which takes account of random sequence generation, allocation concealment, blinding of participants and personnel, blinding of outcome assessment, incomplete outcome data, selective reporting and other biases. Each aspect was graded using three levels (low, unclear or high risk of bias).

### Quality assessment

The certainty level of the result for each outcome of interest was determined with the Grading of Recommendations Assessment, Development and Evaluation (GRADE) tool [23]. This assessed risk of bias, inconsistency of results, indirectness of evidence, imprecision and reporting bias. Certainty was classified as high (no serious concerns found in the five domains), moderate (serious concerns found in one of five domains), low (serious concerns found in two of five domains) or very low (serious concerns found in three or more of five domains).

### Data analysis and synthesis

Meta-analyses were conducted, using Review Manager software (RevMan version 5.4; The Cochrane Collaboration, 2020), if three or more studies with similar interventions investigated the same outcome domain using comparable measures. Standardised mean difference (SMD), 95% confidence intervals (CI) and two-sided *P* values were calculated to measure treatment effects for each outcome. The effect sizes (*d*) were classified using Cohen’s classification, whereby *d* <0.2 was ‘no effect’, *d* = 0.2–0.49 was ‘small’, *d* = 0.5–0.79 was ‘moderate’ and *d* ≥0.8 was ‘large effect’ [24].

For missing data, means and standard deviations (SD) were approximated from available data, such as medians, interquartile ranges and minimum–maximum values using previously reported methods [25–27], and included in meta-analyses unless significant skewness was detected [28]. Standard errors or 95% CIs were converted to SDs using RevMan and Cochrane calculations. If more than one outcome measure was recorded for an outcome in the same study, the most valid and reliable measure was used. Heterogeneity was assessed using the *I*^2^ statistic, where a value between 25 and 50% corresponds to low heterogeneity, 50–75% to moderate heterogeneity and >75% to high heterogeneity. A random-effects model was used, as heterogeneity was expected. Sensitivity analyses were performed when approximated values were used. Funnel plot and Egger’s regression test were used to investigate publication bias [29]. Post hoc secondary analyses to compare non-ICU with ICU patients were performed to explore possible heterogeneity due to this setting and patient group.

## Results

### Study selection

**Figure 1:**
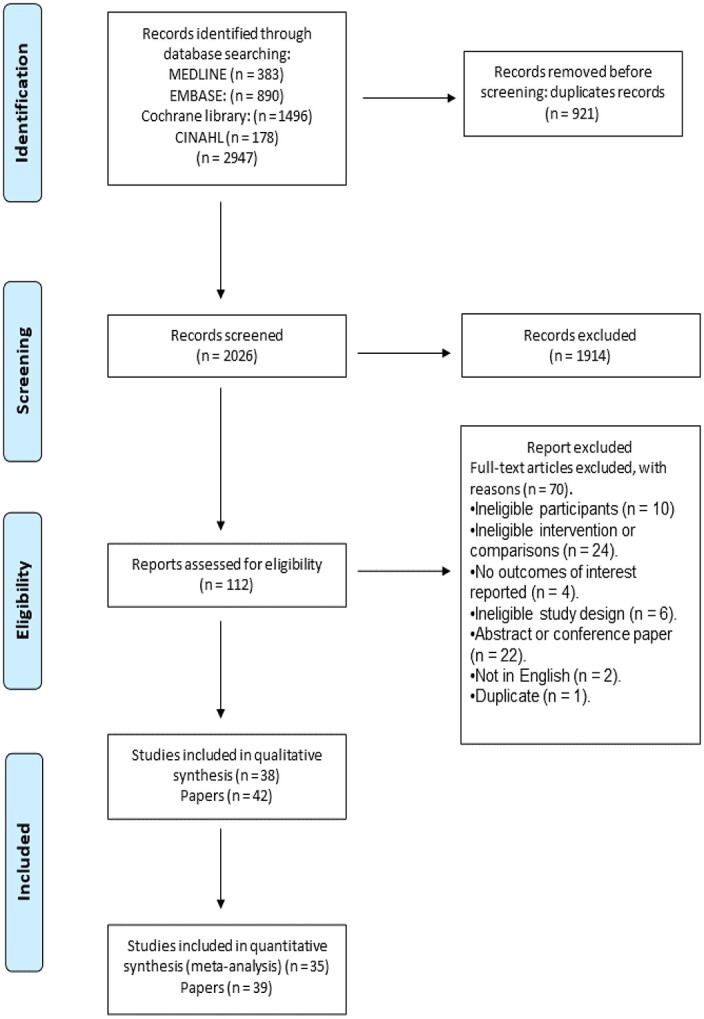
Results of search and study selection process.

The search strategy yielded 2,026 unique titles: 1,914 were excluded based on title and abstract. Of the remaining 112 papers, 70 were excluded on full-text review (supplementary file), leaving 42 eligible papers from 38 studies [30–71]: all 38 studies were included in the qualitative synthesis [30–49, 51–61, 66–71], and 39 papers from 35 studies were included in meta-analyses [30, 31, 33–44, 46–60, 62–71] (Figure 1).

### Characteristics of the studies included

Sample sizes ranged from 6 to 180 participants, with a total sample size of 1,473 participants in all 42 papers. Data from 1,452 participants were used for analysis because in two studies [32, 62] only two of three participant groups were eligible for inclusion. The trials included 1,452 participants but, because study design for 151 participants involved a comparison between their treated and untreated legs, the number of data points for comparison between NMES and control was 1,603.

In total, 894/1,452 (61%) participants were male: two studies included only males [34, 56]. The age range was 19–86 years. Twenty-two studies were conducted in Europe [30, 32–37, 43, 45, 47–51, 55, 56, 58, 59, 61, 62, 66, 70], eight in South America [38–40, 42, 44, 46, 57, 60], five in Asia [41, 53, 54, 67, 68], two in North America [52, 71] and one in Africa [31]. Studies were published between 2003 and 2023. Included studies involved a variety of patient conditions: critical illness [31, 38, 43–45, 47, 49, 52, 54, 58–60]; post-surgery [35–37, 39, 40, 50, 51, 53, 61, 66, 67]; COPD [30, 32, 48, 70]; heart failure [33, 42, 46, 55, 68, 69]; spinal injury [34]; sepsis [56, 57]; COVID-19 [71]; frailty [68]. Table 1 summarises the studies’ characteristics (for full studies characteristics, see supplementary file).

**Table 1:** Characteristics of the included studies

Author	Conditions and setting	*N* (CON/NMES)	Interventions	Outcomes
Abdellaoui, 2011 [30] France	COPD patients **Setting:** ICU	17 (6/9)	Sham vs NMES	Muscle strength Muscle oxidation 6-minute walk test Muscle structure
Abu-khaber, 2013 [31] Egypt	Patients on mechanical ventilation **Setting:** ICU	80 (40/40)	CON (no treatment) vs NMES	Muscle strength
Akar, 2015 [32] Turkey	Patients on mechanical ventilation **Setting:** ICU	20 (10/10)	Mobilisation vs NMES + mobilisation	Muscle strength Mobilisation function Inflammatory response
Arenja, 2021 [33] Switzerland	Acute heart failure old patients **Setting:** Hospital/home	13 (5/4/4)	CON vs low NMES vs high NMES	6-minute walk test Gait speed Health-related QoL
Arija-Blázques, 2014 [34] Spain	Spinal cord injury patients **Setting:** Hospital	8 (3/5)	Sham vs NMES	Muscle CSA Bone markers
Avramidis, 2011 [35] Greece	Patients with total knee arthroplasty **Setting:** Hospital/home	70 (35/35)	CON (PT) vs NMES + PT	Knee function 3-minute walk test QoL
Avramidis, 2003 [36] UK	Patients with total knee arthroplasty **Setting:** Hospital/home	30 (15/15)	CON (PT) vs NMES + PT	Knee pain 3-minute walk test
Braid, 2007 [37] UK	Femoral fracture patients **Setting:** Hospital/home	26 (11/15)	CON (PT) vs NMES + PT	Functional mobility Health-related QoL
Campos, 2022 [38] Brazil	Critically ill patients **Setting:** ICU	74 (40/34)	CON (EM) vs NMES + EM	Functional status Muscle strength Adverse events
Cerqueira, 2018 [39] Brazil	Patients after cardiac valve surgery **Setting:** ICU	59 (33/26)	CON (PT) vs NMES + PT	Walking test Muscle strength Functional independence Health-related QoL
Cerqueira, 2018 [40] Brazil	Patients undergoing cardiac surgery **Setting:** ICU	45 (22/23)	CON (PT) vs NMES + PT	6-minute walking test Lactate level Muscle strength Functional independence
Chen, 2019 [41] Tiawan	Patients undergoing prolonged mechanical ventilation **Setting:** ICU	33 (17/16)	Sham vs NMES	Muscle thickness and circumference Muscle strength Physical function
de Araújo, 2012 [42] Brazil	Heart failure patients **Setting:** Hospital	20 (10/10)	CON (rehabilitation) vs NMES + rehabilitation	6-minute walking test Blood lactate Oxygen saturation
Dirks, 2015 [43] Belgium	Critically ill comatose patients **Setting:** ICU	6 (within subject)	Sham vs NMES	Muscle fibre CSA mRNA and protein expression of selected genes
Falavigna, 2013 [44] Brazil	Patients on mechanical ventilation **Setting:** ICU	11 (within subject)	CON vs NMES	Muscle strength ROM Muscle mass
Fischer, 2016 [45] Austria	Critically ill patients after cardiothoracic surgery **Setting:** ICU	54 (27/27)	Sham vs NMES	Muscle thickness Muscle strength Functional independence
Forestieri, 2017 [46] Brazil	Advanced heart failure patients **Setting:** Hospital	49 (25/24)	CON vs NMES	6-minute walking test
Gerovasili, 2009 [47] Greece	Critically ill patients **Setting:** ICU	26 (13/13)	CON vs NMES	Muscle mass
Giavedoni, 2012 [48] UK	COPD patients **Setting:** Hospital/home	11 (within subject)	CON vs NMES	Muscle strength
Gruther, 2010 [49] Austria	Critically ill patients **Setting:** ICU	33 (17/16)	Sham vs NMES	Muscle thickness
Harbo, 2018 [50] Denmark	Guillain–Barre syndrome **Setting:** Hospital	16 (within subject)	CON vs NMES	Muscle CSA Muscle strength
Hardy, 2022 [51] UK	Patients undergoing abdominal surgery **Setting:** Hospital	15 (within subject)	CON vs NMES	Muscle CSA Muscle thickness Muscle architecture Muscle strength Physical activity level
Kho, 2015 [52] USA	Critically ill patients on mechanical ventilation **Setting:** ICU	34 (18/16)	CON vs NMES	Muscle strength Functional status Maximum walking distance test Hospital LoS
Kitamura, 2019 [53] Japan	Patients after cardiovascular surgery	119 (59/60)	CON vs NMES	Knee muscle strength Concentration of 3-methylhistidine corrected for urinary creatinine 10-minute walk test
Nakanishi, 2020 [54] Japan	Critically ill patients on mechanical ventilation **Setting:** ICU	36 (19/17)	CON (mobilisation) vs NMES + mobilisation	Muscle mass Muscle strength ICU mobility Hospital LoS Amino acid
Poltavskaya, 2022 [55] Russia	Heart failure patients **Setting:** Hospital	45 (23/22)	Sham vs NMES	6-minute walking test QoL Adverse events
Poulsen, 2011 [56] Denmark	Septic shock patients **Setting:** ICU	16 (8/8)	CON vs NMES	Muscle volume
Rodriguez, 2012 [57] Argentina	Septic patients requiring mechanical ventilation **Setting:** ICU	14 (within subject)	CON vs NMES	Arm and leg circumference Biceps thickness Muscle strength
Routsi, 2010 [58] Greece	Critically ill patients **Setting:** ICU	52 (28/24)	CON vs NMES	Muscle strength
Segers, 2021 [59] Belgium	Critically ill patients **Setting:** ICU	47 (within subject)	CON vs NMES	Muscle mass Muscle strength Morphological and molecular markers
Silva, 2019 [60] Brazil	Traumatic brain injury patients on mechanical ventilation **Setting:** ICU	60 (30/30)	CON (PT) + NMES + PT	Muscle architecture Plasma level of systematic inflammation Catabolic responses Hospital LoS
Strasser, 2009 [61] Austria	Patients who underwent abdominal surgery **Setting:** Hospital	18 (within subject)	Sham vs NMES	mRNA level of IGF-1Ea mRNA level of MGF Total RNA content Total protein content Ubiquitin-conjugated proteins Proteasome activity
Suetta, 2004, 2008, 2010 [62–65] Denmark	Patients scheduled for unilateral hip replacement surgery **Setting:** Hospital/home	19 (9/10)	CON vs NMES	Muscle CSA Muscle thickness Muscle strength Hospital LoS Walking test Stair climbing test Sit-to-stand test IGF-I
Sumin, 2020 [66] Russia	Patients with postoperative complications after cardiovascular surgery **Setting:** ICU/hospital	37 (19/18)	CON vs NMES	Knee extensor strength Knee flexor strength Muscle CSA 6-minute walking test
Takino, 2023 [67] Japan	Patients with diabetes after cardiovascular surgery **Setting:** Hospital	180 (90/90)	Sham vs NMES	Knee extensors strength 10-minute walking speed
Tanaka, 2022 [68, 69] Japan	Frail old patients with acute decompensated heart failure **Setting:** Hospital	31 (16/15)	CON (mobilisation) vs NMES + mobilisation	Muscle strength 6-minute walking test Clinical function Adverse events
Vivodtzev, 2006 [70] France	COPD patients **Setting:** Hospital	17 (8/9)	CON vs NMES	QoL Muscle strength Muscle mass 6-minute walking test
Zulbaran-Rojas, 2022 [71] USA	COVID-19 patients **Setting:** ICU	16 (8/8)	Sham vs NMES	Ankle strength Oxygen saturation Safety

*Abbreviation*: N, sample size; CON, control; NMES; neuromuscular electrical stimulation; COPD, chronic obstructive pulmonary disease; ICU, intensive care unit; CSA, cross-sectional area; PT, physiotherapy; EM, early mobilisation; LoS, length of stay; IGF-I, insulin-like growth factor I; IGF-1Ea, insulin-like growth factor-1EA; MGF, mechano growth factor; QoL, quality of life.

### The intervention

Location of the delivery of NMES varied between studies: twenty-one studies were in ICU [30–32, 38–41, 43–45, 47, 49, 52, 54, 56–60, 66, 71], in two of which NMES continued for the remainder of the hospital stay [41, 66]. In seventeen studies, NMES was delivered in hospital wards [33–37, 42, 46, 48, 50, 51, 53, 55, 61, 62, 67, 68, 70], in six of which NMES continued at home after discharge [33, 35–37, 48, 62]. Nine studies used within-subject comparison, comparing one side of the body to the other [43, 44, 48, 50, 51, 56, 57, 59, 61].

Intervention duration differed among studies. Nine studies performed NMES for ≤30 min a day [33, 37, 44, 48, 51, 53, 54, 60, 61], nineteen studies for 30–60 min [30, 31, 34, 38, 41, 43, 45, 47, 50, 52, 56–59, 62, 67, 68, 70, 71] and seven studies for ≥60 min [35, 36, 39, 40, 42, 46, 66]. One study reported progression from 30 to 60 min [49], and another progression from 30 to 90 min [55]. The total period over which NMES was delivered ranged from 3 days to 14 weeks. Frequency of NMES stimulation ranged from 10 to 200 Hz, with pulse duration ranging from 100 to 1,400 μs. Session frequency was 5 days/week in 10 studies [30, 32–34, 37, 38, 41, 49, 55, 68], and 4 days/week in one study [70], whilst the majority of the studies reported daily sessions. Twelve studies reported that NMES was delivered twice per day [35, 36, 39–43, 45, 46, 51, 52, 57].

### Risk-of-bias assessment

**Figure 2:**
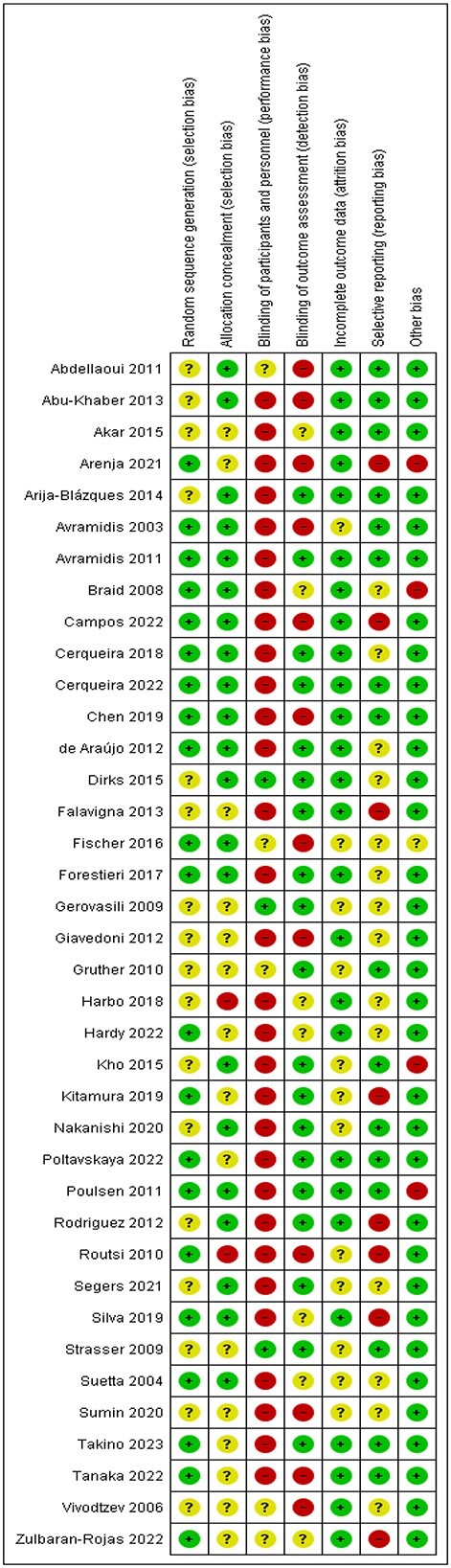
Risk-of-bias summary: review authors’ judgements about each risk-of-bias item for each included study.

Seventeen studies were rated high risk of bias [31, 33, 36–38, 41, 44, 48, 50, 52, 53, 56–58, 60, 66, 68, 69], thirteen with unclear risk of bias [30, 32, 43, 45, 47, 49, 51, 54, 59, 61, 62, 70, 71] and eight low risk of bias [34, 35, 39, 40, 42, 46, 55, 67] (Figure 2). Only 13 studies clearly described both sequence random generation and allocation concealment [35–42, 45, 46, 56, 60, 62]. Few studies achieved blinding of staff providing NMES and participants due to the nature of the intervention. Outcome assessors were blinded in more than half of the studies [34, 35, 39, 40, 42–44, 46, 47, 49, 52–57, 59, 61, 67].

### Muscle strength

**Figure 3:**
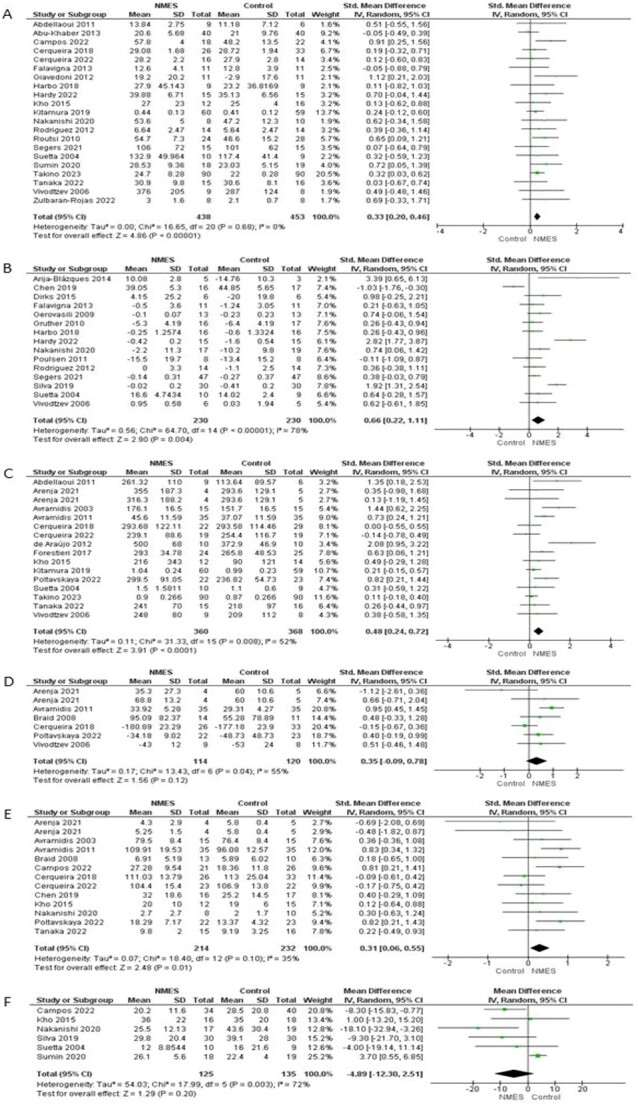
Forest plot: effects of NMES on (A) muscle strength; (B) muscle size; (C) walking performance; (D) health-related QoL; (E) functional mobility; (F) hospital length of stay.

Twenty-four studies reported effect of NMES on muscle strength. Results were pooled from 21 RCTs with 816 participants: one study was excluded because of insufficient data [45] and two studies due to skewness of non-parametric data [32, 41]. The meta-analysis showed a small treatment effect of NMES compared to control (SMD 0.33; 95% CI [0.20, 0.46]; *P* < 0.00001) with no heterogeneity (*I*^2^ = 0%) (Figure 3A). GRADE rating of this small effect was ‘moderate’ certainty (Table 2). Visual inspection of the funnel plot showed nearly symmetrical distribution (Figure S1, supplementary file), and Egger’s regression test showed no significant evidence of asymmetry (*P* > 0.05).

**Table 2:** Summary of findings

**NMES compared to control for hospitalised patients**						
**Patient or population:** Hospitalised patients **Intervention:** NMES **Comparison:** Control						
**Outcomes**	**Anticipated absolute effects** ^**a**^ **(95% CI)**		**Relative effect** **(95% CI)**	**No. of participants** **(studies)**	**Certainty of the evidence** **(GRADE)**	**Comments**
	**Risk with control**	**Risk with NMES**				
Muscle strength		SMD 0.3 SD higher (0.20 higher to 0.46 higher)	–	816 (21 RCTs)	⊕ ⊕ ⊕ ⊝ Moderate^b^	NMES probably results in slight preserving muscle strength
Muscle size		SMD 0.66 SD higher (0.22 higher to 1.11 higher)	–	343 (15 RCTs)	⊕ ⊕ ⊕ ⊝ Moderate^b^	NMES likely results in preserving muscle size
Walking performance		SMD 0.48 SD higher (0.24 higher to 0.72 higher)	–	723 (15 RCTs)	⊕ ⊕ ⊕ ⊝ Moderate^b^	NMES probably increases walking performance
Health-related QoL		SMD 0.35 SD higher (0.09 lower to 0.78 higher)	–	229 (6 RCTs)	⊕ ⊝ ⊝ ⊝ Very low^c^	NMES may result in little to no difference in health-related QoL
Functional mobility		SMD 0.31 SD higher (0.06 higher to 0.55 higher)	–	441 (12 RCTs)	⊕ ⊕ ⊝ ⊝ Low^d^	NMES may result in little to no difference in functional mobility
Hospital length of stay		MD 4.89 Days fewer (12.30 fewer to 2.51 more)	–	260 (6 RCTs)	⊕ ⊝ ⊝ ⊝ Very low^c^	NMES may reduce/have little to no effect on hospital length of stay but the evidence is very uncertain

CI, confidence interval; OR, odds ratio; RR, risk ratio.

GRADE Working Group grades of evidence.

High certainty: we are very confident that the true effect lies close to that of the estimate of the effect.

Moderate certainty: we are moderately confident in the effect estimate: the true effect is likely to be close to the estimate of the effect, but there is a possibility that it is substantially different.

Low certainty: our confidence in the effect estimate is limited: the true effect may be substantially different from the estimate of the effect.

Very low certainty: we have very little confidence in the effect estimate: the true effect is likely to be substantially different from the estimate of effect.

Explanations

^a^The risk in the intervention group (and its 95% CI) is based on the assumed risk in the comparison group and the relative effect of the intervention (and its 95% CI).

^b^Downgraded one level due to risk of bias (blinding was unlikely to be achieved because of the nature of NMES).

^c^Downgraded one level due to risk of bias (blinding was unlikely to be achieved because of the nature of NMES), one level due to inconsistency and one level due to imprecision.

^d^Downgraded one level due to the risk of bias (blinding was unlikely to be achieved because of the nature of NMES) and one level due to inconsistency.

In subgroup analysis, both ICU and non-ICU studies showed a significant benefit of NMES over control. For ICU studies, there was a small effect size (SMD 0.31; 95% CI [0.09, 0.52]; *P* < 0.01) with no heterogeneity (*I*^2^ = 6%). For non-ICU studies, there was also a small effect size (SMD 0.30; 95% CI [0.10, 0.50]; *P* < 0.005) with no heterogeneity (*I*^2^ = 0%) (Figure S2, supplementary file).

### Muscle size

Seventeen studies reported effect of NMES on muscle size. Standardised mean differences were calculated because studies reported different variables (muscle thickness and cross-sectional area (CSA), muscle fibre CSA, arm and leg circumferences, cross-section diameter, muscle volume and muscle mass). One study was not included because of skewed non-parametric data [66] and one because of insufficient data [45]. Fifteen studies including 343 participants were included in the meta-analysis. The meta-analysis showed a significant benefit of NMES over control with high heterogeneity (*I*^2^ = 78%) and moderate effect size (SMD 0.66; 95% CI [0.22, 1.11]; *P* < 0.005, Figure 3B). The GRADE rating of this moderate effect was ‘moderate certainty’ (Table 2).

In subgroup analysis, both ICU and non-ICU studies showed a significant benefit of NMES over control. For ICU studies, there was a moderate effect size (SMD 0.62; 95% CI [0.21, 1.04]; *P* < 0.005) with moderate heterogeneity (*I*^2^ = 66%). For non-ICU studies, there was a large effect size (SMD 1.56; 95% CI [0.05, 3.06]; *P* < 0.05) with high heterogeneity (*I*^2^ = 84%) (Figure S3, supplementary file).

### Function

#### Walking performance

Sixteen studies reported effect of NMES on walking performance, using different measures (3-, 6- and 10-minute walk tests, gait speed and 1,000 feet walking distance). One study was not included because of insufficient data [66]. One study was included as two trials because they used two different NMES techniques [33]. The pooled data from 15 studies including 723 participants showed significant benefit of NMES over control, with a small effect size (SMD 0.48; 95% CI [0.24, 0.72]; *P* < 0.0001, Figure 3C) and moderate heterogeneity (*I*^2^ = 52%). The GRADE rating of this small effect was ‘moderate’ certainty (Table 2).

In subgroup analysis, ICU studies showed a non-significant difference between groups with no heterogeneity (*I*^2^ = 0%) and no/negligible effect (SMD 0.06; 95% CI [−0.31, 0.43]; *P* > 0.05), but in non-ICU settings there was a significant difference with moderate heterogeneity (*I*^2^ = 61%) and a small effect size (SMD 0.49; 95% CI [0.15, 0.83]; *P* < 0.01) (Figure S4, supplementary file).

#### Health-related quality of life

Seven studies reported the effect of NMES on health-related quality of life (HRQoL) using different measures (EuroQoL five dimensions, Short Form 36, Minnesota Living with Heart Failure Questionnaire, Nottingham health profile and the 28-item Maugeri Foundation Respiratory Failure questionnaire). One study was not included because of skewed non-parametric data [38]. One study was included as two trials because they used two different NMES techniques [33]. The pooled data from six studies including 229 participants showed a non-significant benefit of NMES over control group with a small effect size (SMD 0.35; 95% CI [−0.09, 0.78]; *P* > 0.05, Figure 3D) and moderate heterogeneity (*I*^2^ = 55%). The GRADE rating of this small treatment effect was ‘very low’ certainty (Table 2).

#### Functional mobility

Twelve studies reported effect of NMES on activities related to mobility using different measures (Katz Index of Activity of Daily Living, Functional Independence Measure, American Knee Society score, Hospital for Special Surgery Knee-rating score, Functional Status in the ICU, Duke Activity Status Index, Elderly mobility scale, ICU mobility scale and Short Physical Performance Battery). One study was included as two trials because they used two different NMES techniques [33]. The pooled data from 12 studies including 441 participants showed a significant benefit of NMES over control with a small effect size (SMD 0.31; 95% CI [0.06, 0.55]; *P* < 0.05, Figure 3E) and low heterogeneity (*I*^2^ = 35%). The GRADE rating of this small treatment effect was ‘low’ certainty (Table 2).

#### Hospital length of stay

Six studies investigating effect of NMES on hospital length of stay, where NMES was delivered in hospital ward and ICU (260 participants). There was no significant difference between NMES and control groups (mean difference − 4.89 days; 95% CI [−12.30, 2.51]; *P* > 0.05, Figure 3F), with moderate heterogeneity (*I*^2^ = 72%). The GRADE rating for this effect was ‘very low’ certainty (Table 2).

### Molecular and cellular outcomes

The nine studies that examined cellular and molecular biomarkers were not suitable for meta-analysis. We grouped molecular and cellular biomarkers into five categories: fibre type composition; inflammatory mediators; muscle protein synthesis and breakdown; bone; lipid and lipoprotein markers.

Four studies [30, 43, 59, 65] examined muscle fibre type composition. Overall, NMES produced a moderate shift towards fibre type I and a small reduction in type II. However, this evidence was rated to be at low certainty.

Two small studies measured inflammatory mediators. Their results were inconsistent: one [32] showed a reduction in Interleukin 6 (IL-6), whilst the other [60] reported no effect. The study showing a reduction in IL-6 [32] showed no reduction in Interleukin 10 (IL-10) or Tumor necrosis factor alpha (TNF-α).

Five studies investigated muscle protein synthesis and breakdown markers (muscle protein expression [43, 60], mRNA expression [43, 59–61] and 3-methylhistidine concentration corrected for urinary creatinine content [53]) with inconsistent findings. Three studies [43, 53, 60] reported that NMES has no significant effect on muscle protein synthesis and degradation, whereas two other studies [59, 61] reported that NMES had a significant effect on some variables (MyHC-I and proteasome activity) but no effect on others (myofibrillar protein content, MyHC-II and atrogin-1). On the basis that the two studies of moderate quality showed no effect, we judged this to represent evidence of no effect, albeit at low certainty because of risk of bias and inconsistency.

Only one study reported the effect of NMES on bone turnover biomarkers (testosterone, cortisol, growth hormone, insulin-growth factor I, osteocalcin, serum type I collagen C-telopeptide) and lipid and lipoprotein profiles [34]. The study was of low risk of bias, but had a very small sample size (*n* = 8). It found that NMES has no effect on bone, lipid and lipoprotein markers (*P* > 0.05), but in view of the limited sample size, we rated this as ‘inadequate evidence’.

### Adverse events

Twenty-five studies measured adverse events, 13 of which reported no adverse event related to NMES, and 45/553 (9%) participants experienced undesirable experiences (a prickling sensation, hypotension, intolerable stimulation, muscle discomfort, pain and superficial burn).

### Sensitivity analyses

Sensitivity analyses excluding studies using data approximated from non-parametric statistics, studies reporting change score for muscle strength and studies reporting post-intervention score for muscle size produced similar results to the primary analyses for muscle strength, muscle size and walking performance. However, the small significant benefit on functional mobility seen in the primary analysis was not significant in the sensitivity analysis (supplementary file).

## Discussion

In adults hospitalised for a wide range of conditions, NMES produced a small benefit in muscle strength, a moderate benefit in muscle size, a small improvement in walking performance, a small improvement in functional mobility, no effect on HRQoL, no effect on length of stay and inconsistent effects on muscle metabolism. NMES was safe although associated with a small number of minor discomforting symptoms.

Our findings are consistent with previous positive reviews [10, 14, 16–18] of NMES in other populations. There is some discrepancy between our findings and those from reviews of ICU [15, 20] and COPD patients [15, 72]. This inconsistency could be because these reviews were confined to a specific population (ICU and COPD patients) and they included fewer studies (<10 studies) than our review.

These findings show that NMES is a promising intervention to reduce hospital-acquired disability. We were unable to identify an optimal treatment protocol because of the numerous parameters. Further research should identify NMES parameters (electrical stimulation parameters, frequency and duration of NMES) that optimise its effectiveness, convenience and tolerability. Nevertheless, rehabilitation practitioners are justified in offering this intervention for selected individuals, aiming to stimulate as much muscle as possible and as close to maximal contraction as is tolerated, for as long as would be seen in a voluntary exercise programme. Further research should establish the optimal role of this intervention in routine clinical care, including the feasibility of NMES in patient most at risk of hospital-acquired disability such as those with frailty [73]. Treatment packages that blend NMES into best existing rehabilitation practice and train therapists in its use are needed. Future studies should detail the treatment protocols, not only the electrical parameters but also the practical and contextual elements of the intervention, for example by using the TiDIER framework [74].

Our findings are trustworthy because we adhered to PRISMA-P guidelines, valuable because we were able to conduct numerical synthesis (meta-analyses) and robust because the results for our primary outcome (muscle strength) were consistent and stood up to sensitivity analyses. However, there were limitations. Not all studies were at low risk of bias, and most were small, which could have exaggerated the estimated effect sizes. The moderate heterogeneity of the secondary outcomes of muscle size and walking performance may reflect the fact that different studies used different measures of these variables and that our methods could not fully correct for this. The finding of a 5-day reduction in length of hospital stay (an outcome only indirectly related to the direct effects of NMES) had high heterogeneity, and this contributed to our conclusion that this apparent benefit was of low certainty. We excluded studies conducted in languages other than English and although only two studies were excluded on these grounds, this may have reduced the levels of precision of our findings. We did not search unpublished studies although the bias this may have introduced is uncertain.

In conclusion, NMES is a promising technique to contribute to reduction of hospital-acquired disability through improvement or preservation of muscle function, muscle size and functioning.

## Supplementary Material

aa-23-0697-File002_afad236
