# Supplementary material for: The effects of neuromuscular electrical stimulation on hospitalised adults: systematic review and meta-analysis of randomised controlled trials
Source: Age Ageing. 2023 Dec 28;52(12):afad236. doi: 10.1093/ageing/afad236 (PMC10756181; doi:10.1093/ageing/afad236)
Supplement: aa-23-0697-File002_afad236 [file aa-23-0697-file002_afad236.docx]

**The Effects of Neuromuscular Electrical Stimulation (NMES) on hospitalised adults: Systematic Review and Meta-Analysis of Randomised Controlled Trials ̶ Supplementary file**

Table of Contents

[Systematic review search strategy 2](#_Toc148622800)

[List of Excluded studies 6](#_Toc148622801)

[Characteristics of the included studies 12](#_Toc148622802)

[Subgroup analyses 37](#_Toc148622803)

[Funnel plot of muscle strength 40](#_Toc148622804)

[Sensitivity analyses 41](#_Toc148622805)

## Systematic review search strategy

|  | Search Terms |
| --- | --- |
| MEDLINE | 1. Randomized controlled trial.pt. 2. Controlled clinical trial.pt. 3. Randomized.ab. 4. Placebo.ab. 5. Clinical trials as topic.sh. 6. Randomly.ab. 7. Trial.ti. 8. 1 or 2 or 3 or 4 or 5 or 6 or 7 9. exp animals/ not humans.sh. 10. 8 not 9 11. exp adolescent/ or exp adult/ or exp aged/ or exp "aged, 80 and over"/ or exp frail elderly/ or exp middle aged/ or exp young adult/ or exp adolescence/ or exp adult/ or exp adolescent/ 12. (Adolescen* adj2 (people or adult* or person* or patient*)).mp. 13. (Young adj2 (people or adult* or person* or patient*)).mp. 14. (Adult* adj2 (people or adult* or person* or patient*)).mp. 15. (Middle-age* adj2 (people or adult* or person* or patient*)).mp. 16. ((Old* or elder*) adj2 (people or adult* or person* or patient*)).mp. 17. 11 or 12 or 13 or 14 or 15 or 16 18. exp hospitalization/ or exp Patient admission/ or exp Inpatients/ or exp Acute disease/ or exp Critical Illness/ or exp Critical care/ or exp Intensive care units/ or exp hospital admission/ or exp hospital patient/ or exp intensive care/ or ward/ or high dependency unit/ or exp intensive care unit/ or exp acutely ill patient/ or exp critically ill patient/ or exp terminally ill patient/ 19. (Hospital* or Admission* or Inpatient* or admit* or intensive care or ICU or critically ill patient* or critical illness).mp. 20. ((Inpatient* or hospital* or medical or healthcare or ICU or intensive or acute*) adj2 (setting* or admit* or admission* or centre* or center* or ward* or unit* or stay)).mp. 21. 18 or 19 or 20 22. exp Muscles/ or exp Muscle Weakness/ or exp Muscular Atrophy/ or exp Muscle, Skeletal/ 23. ((Musc* adj2 (size or mass or atroph* or cross section* area or CSA or wast* or loss* or weak* or strength or power or force or strong* or function or denervat* or innervat* or biops*)) or (cross section* area or CSA)).mp. 24. 22 or 23 25. exp Immobilization/ or exp Mobility Limitation/ or exp Bed Rest/ or exp Bedridden Persons/ or exp disability/ or exp bed rest/ or exp immobility/ or exp bedridden patient/ 26. (Gait or walk* or disabl* or speed or immobili#ation or immobil* or mobil* or physical perform* or ambulat* or bed-rest or bedrid*).mp. 27. 25 or 26 28. 24 or 27 29. exp Electric stimulation/ or exp electric stimulation therapy/ or exp electrostimulation/ or exp muscle excitation/ or exp nerve stimulation/ or exp stimulation/ or exp stimulus/ or exp neuromuscular electrical stimulation/ or exp transcutaneous electrical nerve stimulation/ 30. (electrotherap* or electric* stimulat* or neuromuscular electrical stimulation or NMES or electromyostimulation or electrostimulation or muscle stimulation).mp. 31. 28 or 29 32. 10 and 17 and 21 and 28 and 31 33. limit 32 to english language |
| EMBASE | 1. exp adolescence/ or exp adult/ or exp adolescent/ 2. (Adolescen* adj2 (people or adult* or person* or patient*)).mp. 3. (Young adj2 (people or adult* or person* or patient*)).mp. 4. (Adult* adj2 (people or adult* or person* or patient*)).mp. 5. (Middle-age* adj2 (people or adult* or person* or patient*)).mp. 6. ((Old* or elder*) adj2 (people or adult* or person* or patient*)).mp. 7. 1 or 2 or 3 or 4 or 5 or 6 8. exp hospitalization/ or exp hospital admission/ or exp hospital patient/ or exp acute disease/ or exp critical illness/ or exp intensive care/ or ward/ or high dependency unit/ or exp intensive care unit/ or exp acutely ill patient/ or exp critically ill patient/ or exp terminally ill patient/ 9. (Hospital* or Admission* or Inpatient* or admit* or intensive care or ICU or critically ill patient* or critical illness).mp. 10. ((Inpatient* or hospital* or medical or healthcare or ICU or intensive or acute*) adj2 (setting* or admit* or admission* or centre* or center* or ward* or unit* or stay)).mp. 11. 8 or 9 or 10 12. exp muscle/ or exp muscle weakness/ or exp muscle atrophy/ or exp skeletal muscle/ 13. ((Musc* adj2 (size or mass or atroph* or cross section* area or CSA or wast* or loss* or weak* or strength or power or force or strong* or function or denervat* or innervat* or biops*)) or (cross section* area or CSA)).mp. 14. 12 or 13 15. exp immobilization/ or exp disability/ or exp bed rest/ or exp immobility/ or exp bedridden patient/ 16. (Gait or walk* or disabl* or speed or immobili#ation or immobil* or mobil* or physical perform* or ambulat* or bed-rest or bedrid*).mp. 17. 15 or 16 18. 14 or 17 19. exp electrostimulation/ or exp electrotherapy/ or exp muscle excitation/ or exp nerve stimulation/ or exp stimulation/ or exp stimulus/ or exp neuromuscular electrical stimulation/ or exp transcutaneous electrical nerve stimulation/ 20. (electrotherap* or electric* stimulat* or neuromuscular electrical stimulation or NMES or electromyostimulation or electrostimulation or muscle stimulation).mp. 21. 19 or 20 22. 7 and 11 and 18 and 21 23. Limit 22 to (human and english language and randomized controlled trial) |
| Cochrane Library | 1. MeSH descriptor: [Adolescent or Adult] explode all trees 2. Adolescen* near/2 (people or adult* or person* or patient*) 3. young near/2 (people or adult* or person* or patient*) 4. adult* near/2 (people or adult* or person* or patient*) 5. middle-age* near/2 (people or adult* or person* or patient*) 6. (old* or elder*) near/2 (people or adult* or person* or patient*) 7. #1 or #2 or #3 or #4 or #5 or #6 8. MeSH descriptor: [Hospitalization or Patient Admission or Inpatients or Acute Disease or Critical Illness or Critical Care or Intensive Care Units] explode all trees 9. (Hospital* or Admission* or Inpatient* or admit* or intensive care or ICU or critically ill patient* or critical illness) 10. ((Inpatient* or hospital* or medical or healthcare or ICU or intensive or acute*) near/2 (setting* or admit* or admission* or centre* or center* or ward* or unit* or stay)) 11. #8 or #9 or #10 12. MeSH descriptor: [Muscles or Muscle, Skeletal or Muscular Atrophy or Muscle Weakness] explode all trees 13. ((Musc* near/2 (size or mass or atroph* or cross section* area or CSA or wast* or loss* or weak* or strength or power or force or strong* or function* or denervat* or innervat* or biops*)) or (cross section* area or CSA)) 14. #12 or #13 15. MeSH descriptor: [Immobilization or Mobility Limitation or Bed Rest or Bedridden Persons] explode all trees 16. (Gait or walk* or disabl* or speed or immobili?ation or immobil* or mobil* or physical perform* or ambulat* or bed-rest or bedrid*) 17. #15 or #16 18. #14 or #17 19. MeSH descriptor: [Electric Stimulation or Electric Stimulation Therapy] explode all trees 20. (electrotherap* or electric* stimulat* or neuromuscular electrical stimulation or NMES or electromyostimulation or electrostimulation or muscle stimulation) 21. #19 or #20 22. #7 and #11 and #18 and #21   in Trials |
| CINAHL | 1. (MH "Adolescence+") OR (MH "Adult+") 2. Adolescen* N2 (people or adult* or person* or patient*) 3. Young N2 (people or adult* or person* or patient*) 4. Middle-age* N2 (people or adult* or person* or patient*) 5. Adult* N2 (people or adult* or person* or patient*) 6. (old* or elder*) N2 (people or adult* or person* or patient*) 7. S1 OR S2 OR S3 OR S4 OR S5 OR S6 8. (MH "Hospitalization+") OR (MH "Patient Admission") OR (MH "Inpatients") OR (MH "Critical Illness") OR (MH "Acute Disease") OR (MH "Critical Care+") OR (MH "Intensive Care Units+") 9. Hospital* or Admission* or Inpatient* or admit* or intensive care or ICU or critically ill patient* or critical illness 10. (Inpatient* or hospital* or medical or healthcare or ICU or intensive or acute*) N2 (setting* or admit* or admission* or centre* or center* or ward* or unit* or stay) 11. S8 OR S9 OR S10 12. (MH "Muscles+") OR (MH "Muscle, Skeletal+") OR (MH "Muscle Weakness+") OR (MH "Muscular Atrophy+") 13. ((Musc*) N2 (size or mass or atroph* or cross section* area or CSA or wast* or loss* or weak* or strength or power or force or strong* or function or denervat* or innervat* or biops*)) or (cross section* area or CSA) 14. S12 OR S13 15. (MH "Immobilization") OR (MH "Immobility") OR (MH "Physical Mobility") OR (MH "Bedridden Persons") 16. Gait or walk* or disabl* or speed or immobili#ation or immobil* or mobil* or physical perform* or ambulat* or bed-rest or bedrid* 17. S15 OR S16 18. S14 OR S17 19. (MH "Electric Stimulation+") OR (MH "Electrical Stimulation, Neuromuscular") 20. electrotherap* or electric* stimulat* or neuromuscular electrical stimulation or NMES or electromyostimulation or electrostimulation or muscle stimulation 21. S19 OR S20 22. S7 AND S11 AND S18 AND S21 23. Limiters - English Language; Human; Randomized Controlled Trials |

## List of Excluded studies

Table S1. Table of excluded studies with reasons

| ID | References | Reason for exclusion |
| --- | --- | --- |
| 1 | Alharbi, A., Womack, E., Marquez, R., & Yarar-Fisher, C. (2022). The Effect of Lower Limb Neuromuscular Electrical Stimulation on Skeletal Muscle Signaling for Glucose Utilization and Systemic Inflammation After Acute Spinal Cord Injury. *Archives of Physical Medicine and Rehabilitation*, *103*(3), e25.‏ | Conference abstract |
| 2 | Alqurashi, H., Gladman, J., Gordon, A., Masud, T., Piasecki, M., Robinson, K., O'Connor, D. (2022). Feasibility of neuromuscular electrical stimulation in fragility fracture patients. European Geriatric Medicine - Volume 13, Issue 0, pp. S93-S94 | Conference abstract |
| 3 | Arellano, D., Aguirre, S., Orellana, D., & Contreras, C. (2019). Effect of Neuromuscular Electrostimulation in the Development of Acquired Weakness in the Intensive Care Unit (ICU-AW) in Adult Patients with Severe Sepsis and Septic Shock in a University Hospital: Results of a Randomized Pilot Study. In *B104. CRITICAL CARE: CATCH-22-BEYOND THE VENTILATOR: THE CHALLENGES OF DELIRIUM, ICU-ACQUIRED WEAKNESS, AND REHABILITATION* (pp. A4117-A4117). American Thoracic Society.‏ | Conference abstract |
| 4 | Au-Yeung, S. S., & Hui-Chan, C. W. (2014). Electrical acupoint stimulation of the affected arm in acute stroke: a placebo-controlled randomized clinical trial. *Clinical rehabilitation*, *28*(2), 149-158.‏ | Ineligible intervention |
| 5 | Azambuja, A. D. C. M., de Almeida Kuhn, A., dos Santos Américo, L., da Silva, M. C., dos Santos, P. P., & dos Santos, L. J. (2018). Neuromuscular electrical stimulation and transcutaneous electrical diaphragmatic stimulation in hospitalized patients with chronic cardiorespiratory diseases: A randomized clinical trial. *Journal of Respiratory and CardioVascular Physical Therapy*, *7*(2), 3-12.‏ | Ineligible intervention |
| 6 | Bao, W., Yang, J., Li, M., Chen, K., Ma, Z., Bai, Y., & Xu, Y. (2022). Prevention of muscle atrophy in ICU patients without nerve injury by neuromuscular electrical stimulation: a randomized controlled study. BMC Musculoskeletal Disorders, 23(1), 1-10.‏ | Ineligible intervention |
| 7 | Baron, M. V., Silva, P. E., Koepp, J., Urbanetto, J. D. S., Santamaria, A. F. M., Dos Santos, M. P., & Pinheiro da Costa, B. E. (2022). Efficacy and safety of neuromuscular electrical stimulation in the prevention of pressure injuries in critically ill patients: a randomized controlled trial. *Annals of Intensive Care*, *12*(1), 53.‏ | Ineligible intervention |
| 8 | Bewarder, M., Klostermann, A., Ahlgrimm, M., Bittenbring, J. T., Pfreundschuh, M., Wagenpfeil, S., & Kaddu-Mulindwa, D. (2019). Safety and feasibility of electrical muscle stimulation in patients undergoing autologous and allogeneic stem cell transplantation or intensive chemotherapy. *Supportive Care in Cancer*, *27*, 1013-1020.‏ | Ineligible study design |
| 9 | Bewarder, M., Klostermann, A., Kaddu-Mulindwa, D., Bittenbring, J., & Pfreundschuh, M. (2017, September). Safety, feasibility and effectiveness of electrical muscle stimulation in patients undergoing autologous and allogeneic stem cell transplantation or chemotherapy requiring hospitalization. In *ONCOLOGY RESEARCH AND TREATMENT* (Vol. 40, pp. 183-183). ALLSCHWILERSTRASSE 10, CH-4009 BASEL, SWITZERLAND: KARGER.‏ | Conference abstract |
| 10 | Busk, H., Kwakkel, G., Skou, S., & Wienecke, T. (2020). Neuromuscular electrical stimulation in addition to exercise therapy in acute ischemic stroke-a randomized clinical trial. International journal of stroke, *15*(1), pp. 221.‏ | Conference abstract |
| 11 | Bustos, M. C., & Lo Presti, M. S. (2020). Effectiveness of pre-surgical neuromuscular electrical stimulation on the recovery time of diaphyseal femoral fractures. *International Journal of Therapy And Rehabilitation*, *27*(8), 1-15.‏ | Ineligible study design |
| 12 | Castellano, J. J., Rojas, A. M., Karia, R., Hunter, T., Slover, J., & Moroz, A. (2016). A randomized, double-blind, placebo-controlled study of neuromuscular electrical stimulation (NMES) use for recovery after elective total hip replacement surgery. *Bulletin of the NYU Hospital for Joint Diseases*, *74*(4), 275.‏ | Ineligible participants |
| 13 | Cebeci, G. C., Cebeci, H., Kucuk, M. P., Kucuk, A. O., Bayrak, I. K., & Ulger, F. (2022). Neuromuscular Electrical Stimulator as a Protective Treatment against Intensive Care Unit Muscle Wasting in Sepsis/Septic Shock Patients. *Journal of the College of Physicians and Surgeons--Pakistan: JCPSP*, *32*(10), 1300-1307.‏ | Ineligible comparisons |
| 14 | Chen, F. C., Shao, H. L., & Han, F. L. (2018). A pilot study of neuromuscular electrical stimulation for neuropathic pain caused by spinal cord injury. *Medicine*, *97*(31).‏ | Ineligible study design |
| 15 | Chen, S., Jiang, Y., Yu, B., Dai, Y., Mi, Y., Tan, Y., ... & Tian, Y. (2019). Effect of transcutaneous neuromuscular electrical stimulation on prevention of intensive care unit-acquired weakness in chronic obstructive pulmonary disease patients with mechanical ventilation. *Zhonghua Wei Zhong Bing Ji Jiu Yi Xue*, *31*(6), 709-713.‏ | Not in English |
| 16 | Dos Santos, F. V., Cipriano Jr, G., Vieira, L., Güntzel Chiappa, A. M., Cipriano, G. B., Vieira, P., & Chiappa, G. R. (2020). Neuromuscular electrical stimulation combined with exercise decreases duration of mechanical ventilation in ICU patients: a randomized controlled trial. *Physiotherapy theory and practice*, *36*(5), 580-588.‏ | Ineligible outcomes |
| 17 | Ely, M. R., Schleifer, G. D., Singh, T. K., Baggish, A. L., & Taylor, J. A. (2022). Exercise training does not attenuate cardiac atrophy or loss of function in individuals with acute spinal cord injury: a pilot study. *Archives of Physical Medicine and Rehabilitation*.‏ | Ineligible intervention |
| 18 | Fossat, G., Baudin, F., Courtes, L., Bobet, S., Dupont, A., Bretagnol, A., & Boulain, T. (2018). Effect of in-bed leg cycling and electrical stimulation of the quadriceps on global muscle strength in critically ill adults: A Randomized Clinical Trial. *Jama*, *320*(4), 368-378.‏ | Ineligible comparisons |
| 19 | França, E. E. T., Gomes, J. P. V., De Lira, J. M. B., Amaral, T. C. N., Vilaça, A. F., Paiva Júnior, M. D. S., & De Castro, C. M. M. B. (2020). Acute effect of passive cycle-ergometry and functional electrical stimulation on nitrosative stress and inflammatory cytokines in mechanically ventilated critically ill patients: a randomized controlled trial. *Brazilian Journal of Medical and Biological Research*, *53*.‏ | Ineligible intervention |
| 20 | Gamez Santiago, A. B., Martinez Caceres, C. M., & Hernández-Morante, J. J. (2022). Effectiveness of Intensively Applied Mirror Therapy in Older Patients with Post-Stroke Hemiplegia: A Preliminary Trial. *European Neurology*, *85*(4), 291-299.‏ | Ineligible comparisons |
| 21 | Glinsky, J., Harvey, L., van Es, P., Chee, S., & Gandevia, S. C. (2009). The addition of electrical stimulation to progressive resistance training does not enhance the wrist strength of people with tetraplegia: a randomized controlled trial. *Clinical rehabilitation*, *23*(8), 696-704.‏ | Ineligible participants |
| 22 | Goll, M., Wollersheim, T., Haas, K., Moergeli, R., Malleike, J., Nehls, F., & Weber-Carstens, S. (2015). Randomised controlled trial using daily electrical muscle stimulation (EMS) in critically ill patients to prevent intensive care unit (ICU) acquired weakness (ICUAW). *Intensive Care Medicine Experimental*, *3*(1), 1-2.‏ | Conference abstract |
| 23 | Gotlin, R. S., Hershkowitz, S., Juris, P. M., Gonzalez, E. G., Scott, W. N., & Insall, J. N. (1994). Electrical stimulation effect on extensor lag and length of hospital stay after total knee arthroplasty. *Archives of physical medicine and rehabilitation*, *75*(9), 957-959.‏ | Ineligible intervention |
| 24 | Greening, N. J., Williams, J. E., Hussain, S. F., Harvey-Dunstan, T. C., Bankart, M. J., Chaplin, E. J., & Steiner, M. C. (2014). An early rehabilitation intervention to enhance recovery during hospital admission for an exacerbation of chronic respiratory disease: randomised controlled trial. *Bmj*, *349*.‏ | Ineligible comparisons |
| 25 | Grunow, J. J., Goll, M., Carbon, N. M., Liebl, M. E., Weber-Carstens, S., & Wollersheim, T. (2019). Differential contractile response of critically ill patients to neuromuscular electrical stimulation. *Critical Care*, *23*(1), 1-12.‏ | Ineligible study design |
| 26 | Grunow, J. J., Wollersheim, T., Carbon, N. M., Kny, M., Giesecke, M., Birchmeier, C., Fielitz, J., Weber-Carstens, S. (2019). Effect of protocol-based physiotherapy and muscle activating measures on muscle synthesis and degradation balance in intensive care unit acquired weakness. Intensive Care Medicine Experimental, *0*(0).‏ | Conference abstract |
| 27 | Harbor, T., Markvardsen, L., Hellfritzsch, M., & Andersen, H. (2018). Neuromuscular electrical stimulation in the acute and subacute rehabilitation of Guillain Barre Syndrome. In *JOURNAL OF THE PERIPHERAL NERVOUS SYSTEM* (Vol. 23, No. 4, pp. 330-330). 111 RIVER ST, HOBOKEN 07030-5774, NJ USA: WILEY.‏ | Conference abstract |
| 28 | Hausladen, R., Hering, D., Haymore, J., Judd, G., Sanchez, S., & Badjatia, N. (2019). Safety and feasibility of neuromuscular electrical stimulation and high-protein supplementation acutely after SAH (nct03201094).Neurocrit Care, 31:S1–S341.‏ | Conference abstract |
| 29 | Homma, M., Miura, M., Hirayama, Y., Takahashi, T., Miura, T., Yoshida, N., & Ebihara, S. (2022). Belt Electrode-Skeletal Muscle Electrical Stimulation in Older Hemodialysis Patients with Reduced Physical Activity: A Randomized Controlled Pilot Study. *Journal of Clinical Medicine*, *11*(20), 6170.‏ | Ineligible participants |
| 30 | Iwatsu k., Effect of neuromuscular electrical stimulation on frailty in patients with acute decompensated heart failure. Available from: https://center6.umin.ac.jp/cgi-open-bin/ctr_e/ctr_view.cgi?recptno=R000037544 | Ineligible outcomes  (protocol only) |
| 31 | Kiryu K., The effectiveness of electrical muscle stimulation in patients after open heart surgery. Available from: https://center6.umin.ac.jp/cgi-open-bin/ctr_e/ctr_view.cgi?recptno=R000045532 | Ineligible outcomes  (protocol only) |
| 32 | Klika, A. K., Yakubek, G., Piuzzi, N., Calabrese, G., Barsoum, W. K., & Higuera, C. A. (2022). Neuromuscular electrical stimulation use after total knee arthroplasty improves early return to function: a randomized trial. *The Journal of Knee Surgery*, *35*(01), 104-111.‏ | Ineligible participants |
| 33 | Lee, C. K. (2018). Preserving the limbs muscle bulk for patients with critical illness on mechanical ventilation in ICU.‏ Available from: https://www.chictr.org.cn/showproj.html?proj=17834 | Ineligible outcomes  (protocol only) |
| 34 | Lo Re, V., Russelli, G., Bulati, M., Iannolo, G., Martucci, G., Panarello, G., Lo Gerfo, E., Avorio, F., Sparacia, G., Parla, G., et al. (2022). COGNITIVE OUTCOMES IN PATIENTS TREATED WITH POST-OPERATIVE ELECTRICAL STIMULATION AFTER CORONARY ARTERY BYPASS GRAFT (CABG). Neurological sciences - Volume 43, Issue 0, pp. S423‐S424 | Conference abstract |
| 35 | Lopez-Lopez, L., Torres-Sanchez, I., Rodriguez-Torres, J., Cabrera-Martos, I., Ortiz-Rubio, A., & Valenza, M. C. (2019). Does adding an integrated physical therapy and neuromuscular electrical stimulation therapy to standard rehabilitation improve functional outcome in elderly patients with pneumonia? A randomised controlled trial. *Clinical Rehabilitation*, *33*(11), 1757-1766.‏ | Ineligible comparisons |
| 36 | Maddocks, M., Halliday, V., Chauhan, A., Taylor, V., Nelson, A., Sampson, C., & Wilcock, A. (2013). Neuromuscular electrical stimulation of the quadriceps in patients with non-small cell lung cancer receiving palliative chemotherapy: a randomized phase II study. *PloS one*, *8*(12), e86059.‏ | Ineligible participants |
| 37 | Maddocks, M., Lewis, M., Chauhan, A., Manderson, C., Hocknell, J., & Wilcock, A. (2009). Randomized controlled pilot study of neuromuscular electrical stimulation of the quadriceps in patients with non-small cell lung cancer. *Journal of pain and symptom management*, *38*(6), 950-956.‏ | Ineligible participants |
| 38 | Maggioni, M. A., Cè, E., Giordano, G., Bertoli, S., Battezzati, A., Veicsteinas, A., & Merati, G. (2012). Effects on body composition of different short-term rehabilitation programs in long-stay hospitalized elderly women. *Aging clinical and experimental research*, *24*, 619-626.‏ | Ineligible participants |
| 39 | Maggioni, M. A., Cè, E., Rampichini, S., Ferrario, M., Giordano, G., Veicsteinas, A., & Merati, G. (2010). Electrical stimulation versus kinesitherapy in improving functional fitness in older women: a randomized controlled trial. *Archives of gerontology and geriatrics*, *50*(3), e19-e25.‏ | Ineligible participants |
| 40 | Mateo, A. C., López, L. L., Torres, J. R. R., Sáncehz, I. T., Santiago, M. G., Núñez, J. M., & Valenza, M. C. (2021). Comparison of two physiotherapy treatments in patients with acute exacerbation of chronic obstructive pulmonary disease: a pilot study.‏ European Respiratory Journal - Volume 58: PA3911; DOI: 10.1183/13993003.congress-2021.PA3911 | Conference abstract |
| 41 | Mateo, A. C., López, L. L., Torres, J. R. R., Sánchez, I. T., Santiago, M. G., Valero, A. C., & Valenza, M. C. (2021). Effects of a NMES program in exercise capacity of hospitalized severely hypoxemic COPD patients.‏ European respiratory journal - Volume 58: PA317; DOI: 10.1183/13993003.congress-2021.PA317 | Conference abstract |
| 42 | Matsuse, H., Nago, T., Shinozaki, N., Hashida, R., Takano, Y., & Shiba, N. (2017). Combined Application of Electrical Stimulation and Volitional Contraction Prevents Muscle Weakness. *Archives of Physical Medicine and Rehabilitation*, volume 98 (issue 10), page e45.‏ DOI: https://doi.org/10.1016/j.apmr.2017.08.138 | Conference abstract |
| 43 | Micah Drummond. (2015). ClinicalTrials.gov [Internet]. National Library of Medicine (US). Identifier NCT02566590, Preventing the Loss of Muscle and Function in Hospitalized Older Adults; 2015 Oct 2. Available from: https://classic.clinicaltrials.gov/ct2/show/NCT02566590 | Ineligible outcomes  (protocol only) |
| 44 | Nakamura, K., Kihata, A., Naraba, H., Kanda, N., Takahashi, Y., Sonoo, T., & Morimura, N. (2019). Efficacy of belt electrode skeletal muscle electrical stimulation on reducing the rate of muscle volume loss in critically ill patients: A randomized controlled trial. *Journal of Rehabilitation Medicine*, *51*(9), 705-711.‏ | Ineligible comparisons |
| 45 | Nakanishi, N., Oto, J., Tsutsumi, R., Yamamoto, T., Ueno, Y., Nakataki, E., & Nishimura, M. (2019). Electrical muscle stimulation on upper and lower limb muscle in critically ill patients. Intensive Care Medicine Experimental(2019) , 7(Suppl 3) : 55 | Conference abstract |
| 46 | Nakanishi, N., Oto, J., Tsutsumi, R., Yamamoto, T., Ueno, Y., Nakataki, E., & Nishimura, M. (2020). Effect of electrical muscle stimulation on upper and lower limb muscles in critically ill patients: a two-center randomized controlled trial. *Critical care medicine*, *48*(11), e997-e1003.‏ | Duplicate |
| 47 | Nozoe, M., Kanai, M., Kubo, H., Takeuchi, Y., Kobayashi, M., Yamamoto, M., & Mase, K. (2017). Efficacy of neuromuscular electrical stimulation for preventing quadriceps muscle wasting in patients with moderate or severe acute stroke: A pilot study. *NeuroRehabilitation*, *41*(1), 143-149.‏ | Ineligible study design |
| 48 | Oleinik, P., Sumin, A. N., & Bezdenezhnykh, A. V. (2021). Effects of the use neuromuscular electrical stimulation of the lower extremities in the prehabilitation of patients in a before cardiac surgery. *European Journal of Preventive Cardiology*, *28*(Supplement_1), zwab061-365.‏ | Conference abstract |
| 49 | Oleinik, P., Sumin, A. N., & Bezdenezhnykh, A. V. (2021). Neuromuscular electrical stimulation in early rehabilitation of patients with postoperative complications after cardiovascular surgery. *European Journal of Preventive Cardiology*, *28*(Supplement_1), zwab061-363.‏ | Conference abstract |
| 50 | Pandey, D. P. (2018). Exploring prevention and treatment strategy for critical illness associated neuromuscular weakness. Neurorehabilitation and Neural Repair - Volume 0, Issue 0, pp. 10th World Congress for NeuroRehabilitation, WCNR 2018. Mumbai India. 32 (4-5) (pp 459) | Conference abstract |
| 51 | Patsaki, I., Gerovasili, V., Sidiras, G., Karatzanos, E., Mitsiou, G., Papadopoulos, E., & Nanas, S. (2017). Effect of neuromuscular stimulation and individualized rehabilitation on muscle strength in intensive care unit survivors: a randomized trial. *Journal of critical care*, *40*, 76-82.‏ | Ineligible comparisons |
| 52 | Péran, L., Beaumont, M., Le Ber, C., Le Mevel, P., Berriet, A. C., Nowak, E., & Couturaud, F. (2022). Effect of neuromuscular electrical stimulation on exercise capacity in patients with severe chronic obstructive pulmonary disease: A randomised controlled trial. *Clinical Rehabilitation*, *36*(8), 1072-1082.‏ | Ineligible comparisons |
| 53 | Pinto, N., Santana, L., Xavier, A., & Maldaner, V. (2016). Effects of neuromuscular electrical stimulation in critically ill patients by assessment with peak torque evoked: A randomized controlled trial.‏ European Respiratory Journal, 48: PA4421; | Conference abstract |
| 54 | Quittan, M., Wiesinger, G. F., Sturm, B., Puig, S., Mayr, W., Sochor, A., & Fialka-Moser, V. (2001). Improvement of thigh muscles by neuromuscular electrical stimulation in patients with refractory heart failure: a single-blind, randomized, controlled trial. *American journal of physical medicine & rehabilitation*, *80*(3), 206-214.‏ | Ineligible participants |
| 55 | Reidel, L. T., Cecchele, B., Sachetti, A., & Calegari, L. (2020). Effects of neuromuscular electrostimulation of quadriceps on the functionality of fragileand pre-frail hospitalized older adults: randomized clinical trial. *Fisioterapia e Pesquisa*, *27*, 126-132.‏ | Ineligible intervention |
| 56 | Rosewilliam, S., Malhotra, S., Roffe, C., Jones, P., & Pandyan, A. D. (2012). Can surface neuromuscular electrical stimulation of the wrist and hand combined with routine therapy facilitate recovery of arm function in patients with stroke?. *Archives of physical medicine and rehabilitation*, *93*(10), 1715-1721.‏ | Ineligible intervention |
| 57 | Sanchez, I. T., Mateo, A. C., López, L. L., Ciuró, A. H., Rubio, A. O., & Valenza, M. C. (2020). Feasibility of a NMES program in hypoxic severe hospitalized patients with COPD.‏ European Respiratory Journal, 56: 3020 | Conference abstract |
| 58 | Teschler, M., Heimer, M., Schmitz, B., Kemmler, W., & Mooren, F. C. (2021). Four weeks of electromyostimulation improves muscle function and strength in sarcopenic patients: a three‐arm parallel randomized trial. *Journal of Cachexia, Sarcopenia and Muscle*, *12*(4), 843-854.‏ | Ineligible intervention |
| 59 | Timofte, I., Wells, C., Hersi, K., Ryan, A., Varghese, A. M., Vesselinov, R., & Verceles, A. (2021). Nutritional Supplementation and Neuromuscular Electrical Stimulation in Lung Transplant Patients. *The Journal of Heart and Lung Transplantation*, *40*(4), S359-S360.‏ | Conference abstract |
| 60 | Varghese, A., Ryan, A., Wells, C., Li, G., Baer, D., Parker, E. A., ... & Timofte, I. (2022). Post-Transplant Metabolomics Profiles in Patients Undergoing Lung Transplantation. *The Journal of Heart and Lung Transplantation*, *41*(4), S51.‏ | Conference abstract |
| 61 | Verceles, A. C., Serra, M., Davis, D., Alon, G., Wells, C. L., Parker, E., & Terrin, M. L. (2023). Combining exercise, protein supplementation and electric stimulation to mitigate muscle wasting and improve outcomes for survivors of critical illness—The ExPrES study. *Heart & Lung*, *58*, 229-235.‏ | Ineligible comparisons |
| 62 | Vieira, P. J., Chiappa, A. M. G., Cipriano Jr, G., Umpierre, D., Arena, R., & Chiappa, G. R. (2014). Neuromuscular electrical stimulation improves clinical and physiological function in COPD patients. *Respiratory Medicine*, *108*(4), 609-620.‏ | Ineligible participants |
| 63 | Wappel, S. R., Ali, O., Serra, M., Wells, C. L., Davis, D., Alon, G., & Verceles, A. C. (2017). The Effect Of An Exercise, Nutrition And Neuromuscular Electrical Stimulation Intervention On Acute Muscle Wasting In Critically Ill Patients Receiving Mechanical Ventilation. In *A104. CRITICAL CARE: IMPROVING ICU EXERCISE, REHABILITATION, RECOVERY, AND SURVIVORSHIP* (pp. A2747-A2747). American Thoracic Society.‏ | Conference abstract |
| 64 | Yamada S. et al., Effects of Postoperative Electrical Muscle Stimulation on Functional Decline in Patients after Cardiovascular Surgery - Multicentre Randomized Controlled Trial – Available from: https://center6.umin.ac.jp/cgi-open-bin/ctr_e/ctr_view.cgi?recptno=R000013077 | Ineligible outcomes  (protocol only) |
| 65 | Yen, H. C., Chen, W. S., Jeng, J. S., Luh, J. J., Lee, Y. Y., & Pan, G. S. (2019). Standard early rehabilitation and lower limb transcutaneous nerve or neuromuscular electrical stimulation in acute stroke patients: a randomized controlled pilot study. *Clinical Rehabilitation*, *33*(8), 1344-1354.‏ | Ineligible intervention |
| 66 | Yoshida, Y., Ikuno, K., & Shomoto, K. (2017). Comparison of the effect of sensory-level and conventional motor-level neuromuscular electrical stimulations on quadriceps strength after total knee arthroplasty: a prospective randomized single-blind trial. *Archives of Physical Medicine and Rehabilitation*, *98*(12), 2364-2370.‏ | Ineligible intervention |
| 67 | Youssef, M. (2022). Neuromuscular electrical stimulation and exercises effect on functional exercise performance and quality of life in cases of liver cirrhosis. *Physiotherapy Quarterly*, *30*(3), 1-6.‏ | Ineligible comparisons |
| 68 | Zanotti, E., Felicetti, G., Maini, M., & Fracchia, C. (2003). Peripheral muscle strength training in bed-bound patients with COPD receiving mechanical ventilation: effect of electrical stimulation. *Chest*, *124*(1), 292-296.‏ | Ineligible intervention |
| 69 | Zhu, C., Liu, B., Yang, T., Mei, Q., Pan, A., & Zhao, D. (2018). Effect of early rehabilitation physiotherapy on muscle quality and function in critically ill patients. *Zhonghua wei Zhong Bing ji jiu yi xue*, *30*(6), 569-572.‏ | Not in English |
| 70 | Zinglersen, A. H., Halsteen, M. B., Kjaer, M., & Karlsen, A. (2018). Can electrical stimulation enhance effects of a functional training program in hospitalized geriatric patients?. *Experimental gerontology*, *106*, 101-108.‏ | Ineligible intervention |

## Characteristics of the included studies

Table 2. Characteristics of the included studies

| Author | Conditions and setting | Groups (n), age and ethnicity | NMES intervention | | | Outcomes | Report source of funding |
| --- | --- | --- | --- | --- | --- | --- | --- |
|  |  |  | **Training protocol** | **NMES parameters** | **Stimulated muscles** |  |  |
| Abdellaoui et al  2011  France | COPD patients | NMES (9)  **Age, median (IQR):** 59 (57-69) | 1h/day  Once/day  5 days/week  6 weeks (30h) | **Frequency:** 35 Hz  **Pulse width:** 400 µs  **Intensity:** maximum tolerated  **Duty cycle:** constant current  **Waveform:** biphasic symmetric | Quadriceps and hamstring of both legs | **Primary:**   - Muscle strength (MVC) - Muscle oxidation (MHC)   **Secondary:**   - Pulmonary function (FEV_1_, FVC and FEV_1_/FVC ratio). - 6-minute walk test - Muscle structure (oxidation stress and MHC fibre composition) | Yes (non-commercial funder) |
|  | **Setting:** ICU | Control (Sham) (6)  **Age, median (IQR):** 67 (59-72)  Ethnicity: not reported |  |  |  |  |  |
| Abu-Khaber et al  2013  Egypt | Patients on mechanical ventilation | NMES (40)  **Age:** 59.07 ± 5.32 | 1h/day (5 min warm up and recovery)  Once/day  Daily  Until ICU discharge | **Frequency:** 50 Hz  **Pulse width:** 200 µs  **Intensity:** visible or palpable contraction  **Duty cycle:** 15s On (Off not reported)  **Waveform:** biphasic symmetric | Quadriceps of both legs | **Primary:**   - Muscle strength   **Secondary:**  None | No |
|  | **Setting:** ICU | Control (no treatment) (40)  **Age:** 57.57 ± 6.80 |  |  |  |  |  |
| Akar et al  2015  Turkey | Patients on mechanical ventilation | NMES + limb mobilisation (10)  **Age:** 70 ± 12.28  Ethnicity: not reported | Per day (not reported)  5days/week  4 weeks (20 session) | **Frequency:** 50 Hz  **Pulse width:** not reported  **Intensity:** visible or palpable contraction  **Duty cycle:** 6s On (Off not reported)  **Waveform:** biphasic symmetric, square waves | Deltoid and quadriceps- bilaterally | **Primary:**   - Manual muscle strength - Mobilization Function. - Inflammatory response (CRP, IL-6, IL-8, IL-10 and TNF-a)   **Secondary:**  None | No |
|  | **Setting:** ICU | Control (limb mobilisation) (10)  **Age:** 68.00 ± 17.77  Ethnicity: not reported |  |  |  |  |  |
| Arenja et al  2021  Switzerland | Acute heart failure old patients | Low NMES (4)  **Age:** 76 ± 7.6  High NMES (4)  **Age:** 78.8 ± 7.4 | 30 min/day  5 days/week  6 weeks | **Frequency:**  Low NMES: 25 Hz  High NMES: 50 Hz  **Pulse width:**  Low NMES: 1 ms Hz  High NMES: 10 msec  **Intensity:** not reported  **Duty cycle:**  Low NMES: 2/5s  High NMES: not reported  **Waveform:**  Low NMES: not reported  High NMES: rectangular impulses | Femoral and tibial muscles of both legs | **Primary:**   - 6-minute walk test   **Secondary:**   - Gait speed - TUG - Isometric force measurements - Cardiac function - Health-related QoL | Yes (non-commercial funder) |
|  | **Setting:** Hospital/Patient home | Control (Placebo) (5)  **Age:** 83.9 ± 10.5  Ethnicity: not reported |  |  |  |  |  |
| Arija-Blázques et al  2014  Spain | Spinal cord injury patients | NMES (5)  **Age:** 41.7 ± 12. | 47min/day  Once/day  5 days/week  14 weeks | **Frequency:** 30 Hz  **Pulse width:** 200 µs  **Intensity:** visible contraction  **Duty cycle:** not reported  **Waveform:** not reported | Quadriceps of both legs | **Primary:**   - Muscle CSA. - Bone markers ( total testosterone, cortisol, growth hormone, insulin-growth factor I, osteocalcin, serum type I collagen C-telopeptide). - Lipid, and lipoprotein profiles   **Secondary:**  None | No |
|  | **Setting:** Hospital | Control (Sham) (3)  **Age:** 36.00 ± 13.6  Ethnicity: not reported |  |  |  |  |  |
| Avramidis et al  2011  Greece | Patients with total knee arthroplasty | NMES + PT (35)  **Age**: 70.54 ± 4.68. | 2h/session Twice/day  Daily  6 weeks | **Frequency:** 40 Hz  **Pulse width:** 300 µs  **Intensity:** maximum tolerated  **Duty cycle:** 8/8s  **Waveform:** not reported | Vastus medialis of both legs | **Primary:**   - Knee function - 3-minute walking test - QoL   **Secondary:**  None | No |
|  | **Setting**: Hospital/Patient home | Control (PT) (35)  **Age**: 70.66 ± 3.37  Ethnicity: not reported |  |  |  |  |  |
| Avramidis et al  2003  UK | Patients with total knee arthroplasty | NMES + PT (15)  **Age**: 68.20 ± 10.59. | 2h/session Twice/day  Daily  6 weeks | **Frequency:** 40 Hz  **Pulse width:** 300 µs  **Intensity:** maximum tolerated  **Duty cycle:** 8/8s  **Waveform:** biphasic asymmetric. | Vastus medialis of both legs | **Primary:**   - 3-minute walking test - Energy cost of exercise - Knee pain   **Secondary:**  None | No |
|  | **Setting**: Hospital/Patient home | Control (PT) (15)  **Age**: 71.20 ± 7.83  Ethnicity: not reported |  |  |  |  |  |
| Braid et al  2007  UK | Femoral fracture patients | NMES + usual PT  (15)  **Age:** 81 ± 7.7 | 18 min/day  Once/day  5 days/week as inpatient and 2 days/week after discharge  For 6 weeks | **Frequency:** 40, 60, 80, and 100 Hz  **Pulse width:** not reported  **Intensity:** patient tolerance  **Duty cycle: 7**/23s  **Waveform:** not reported | Quadriceps of the fractured leg | **Primary:**   - Change in leg extensor power   **Secondary:**   - Functional mobility - Disability - Health-related QoL | Yes (non-commercial funder) |
|  | **Setting:** Hospital/Patient home | Control (usual PT)  (11)  **Age:** 80 ± 6.6  Ethnicity: not reported |  |  |  |  |  |
| Campos et al  2022  Brazil | Critically ill patients | NMES + EM (34)  **Age**: 42.50 ± 14.90 | 60 min/day  Once/day  5 days/week  Until ICU discharge | **Frequency:** 80 Hz  **Pulse width:** 400 µs  **Intensity:** palpable or visible contraction  **Duty cycle:** 5/10s  **Waveform:** biphasic symmetrical impulses | Quadriceps and tibialis anterior of both legs | **Primary:**   - Functional status at ICU   **Secondary:**   - Muscle strength - Functional status (Physical Function Test in ICU) - Adverse events | Yes (non-commercial funder) |
|  | **Setting:** ICU | Control (EM) (40)  **Age**: 46.70 ± 17.90  Ethnicity: not reported |  |  |  |  |  |
| Cerqueira et al  2018  Brazil | Patients after cardiac valve surgery | NMES + usual PT  (26)  **Age:** 41.80 ± 13.17 | 60 min/session  Twice/day  Daily  For 5 days (10 sessions) | **Frequency:** 50 Hz  **Pulse width:** 400 µs  **Intensity:** palpable or visible contraction  **Duty cycle:** 3/9s  **Waveform:** not reported | Quadriceps and gastrocnemius of both legs | **Primary:**   - Walking (ambulation) test   **Secondary:**   - Muscle strength - Functional independence - Health-related QoL | Yes (non-commercial funder) |
|  | **Setting:** ICU | Control (usual PT)  (33)  **Age:** 42.21 ± 14.36  Ethnicity: not reported |  |  |  |  |  |
| Cerqueira et al  2022  Brazil | Patients undergoing cardiac surgery | NMES + PT (23)  **Age**: 47.80 ± 13.90 | 60 min/session  Twice/day  Daily  For 5 days (10 sessions) | **Frequency:** 50 Hz  **Pulse width:** 400 µs  **Intensity:** palpable or visible contraction  **Duty cycle:** 3/9s  **Waveform:** not reported | Rectus femoris and gastrocnemius of both legs | **Primary:**   - 6-minute walking test   **Secondary:**   - 10-minute gait speed - Lactate level - Muscle strength - Electromyographic activity - Functional independence | Yes (non-commercial funder) |
|  | **Setting:** ICU | Control (PT) (22)  **Age**: 46.40 ± 13.50  Ethnicity: not reported |  |  |  |  |  |
| Chen et al  2019  Tiawan | Patients undergoing prolonged mechanical ventilation | NMES  (16)  **Age:** 77.7 ± 14.3 | 30 min/session  Twice/day  5 days/week  For 2 weeks | **Frequency:** 50 Hz  **Pulse width: 4**00 µs  **Intensity:** visible contraction  **Duty cycle:** 2/4s  **Waveform:** biphasic waves | Quadriceps of both legs | **Primary:**   - Pulmonary function - Muscle function (thickness, circumference, and strength) - Physical function   **Secondary:**   - Hospitalisation outcomes (survival status, weaning outcomes, ventilation duration, and ICU length of stay | Yes (non-commercial funder) |
|  | **Setting:** ICU | Control (sham)  (17)  **Age:**  73.8 ± 17.8  Ethnicity: not reported |  |  |  |  |  |
| de Araújo et al  2012  Brazil | Heart failure patients | NMES + rehabilitation (10)  **Age**: 52.2 ± 9 | 1h/session  Twice/day  Daily  Until discharge | **Frequency:** 20 Hz  **Pulse width:** 200 µs  **Intensity:** not reported  **Duty cycle:** 20/20s  **Waveform:** biphasic symmetric, rectangular pulses | Rectus femoris of both legs | **Primary:**   - 6-minute walking test   **Secondary:**   - Venous blood lactate and venous saturation of oxygen | No |
|  | **Setting**: Hospital | Control (rehabilitation) (10)  **Age**: 49.5 ± 14.3  Ethnicity: not reported |  |  |  |  |  |
| Dirks et al  2015  Belgium | Critically ill comatose patients | NMES (6)  **Within subject comparison**  Age: 63.3 ± 17 | 30/session  Twice/day  Daily  Until end of sedation | **Frequency:** 100 Hz (5 Hz for warm up and recovery)  **Pulse width:** 400 µs (250 µs for warm up and recovery  **Intensity:** visible and palpable contraction (progressive every 3 min).  **Duty cycle:** 5/10s  **Waveform:** biphasic symmetric, rectangular pulses | Rectus femoris and vastus lateralis – unilaterally | **Primary:**   - Muscle fibre type specific CSA - mRNA & protein expression of selected genes (FOXO1, MAFbx, MuRF1, mTOR, P70S6K).   **Secondary:**  None | Yes (no funding received) |
|  | **Setting**: ICU | Control (Sham) (6)  **Within subject comparison**  **Age**: 63.3 ± 17  Ethnicity: not reported |  |  |  |  |  |
| Falavigna et al  2013  Brazil | Patients on mechanical ventilation | NMES (11)  **Within subject comparison**  **Age**: 34 ± 17.3 | 20 min (for each muscle)/day  Daily  Until patient attained a force of 4 on the scale of muscle strength | **Frequency:** 50 Hz  **Pulse width:** 400 µs  **Intensity:** visible or palpable contraction  **Duty cycle:** 9/9s  **Waveform:** biphasic symmetric. | Quadriceps and tibialis anterior – unilaterally | **Primary:**   - Muscle strength - ROM of ankle - Muscle mass (thigh and leg circumferences).   **Secondary:**  None | No |
|  | **Setting**: ICU | Control (passive mobilisation) (11)  **Within subject comparison**  **Age**: 34 ± 17.3  Ethnicity: not reported |  |  |  |  |  |
| Fischer et al  2016  Austria | Critically ill patients after cardiothoracic surgery | NMES (27)  **Age**: 63.3 ± 15.5 | 30 min/session  Twice/day  Daily  During ICU stay but no longer than 14 days | **Frequency:** 66 Hz  **Pulse width:** 400 µs  **Intensity:** visible or palpable contraction  **Duty cycle:** 3.5/4.5 s  **Waveform:** biphasic, rectangular pulses | Quadriceps of both legs | **Primary:**   - Muscle layer thickness - Muscle strength.   **Secondary:**   - Functional Independence Measure - TUG - Grip strength - patient satisfaction | Yes (non-commercial funder) |
|  | **Setting**: ICU | Control (Sham) (27)  **Age**: 69.7 ± 13.1  Ethnicity: not reported |  |  |  |  |  |
| Forestieri et al  2017  Brazil | Advanced heart failure patients | NMES (24)  **Age**: 52.58 ±14.71 | 1h (5min warm up and recovery)/session  Twice/day  During hospital stay (around 2 weeks) | **Frequency:** 40 Hz  **Pulse width:** 400 µs  **Intensity:** maximum tolerated to attained visible contraction  **Duty cycle:** 10/20s  **Waveform:** biphasic current | Quadriceps and calf muscles of both legs | **Primary:**   - 6-minute walking test   **Secondary:**  None | Yes (no funding received) |
|  | **Setting**: Hospital | Control (UR) (25)  **Age**: 51.52 ± 11.03  Ethnicity: not reported |  |  |  |  |  |
| Gerovasili et al  2009  Greece | Critically ill patients | NMES (13)  **Age**: 59 ± 23 | 55 min (5min warm up & recovery)/session  Once/day  Daily  8 days | **Frequency:** 45 Hz  **Pulse width:** 400 µs  **Intensity:** visible or palpable contraction  **Duty cycle:** 12/6s  **Waveform:** biphasic symmetric | Quadriceps and peroneus longus of both legs | **Primary:**   - Muscle mass   **Secondary:**  None | Yes (non-commercial funder) |
|  | **Setting**: ICU | Control (no treatment) (13)  **Age**: 56 ± 19  Ethnicity: not reported |  |  |  |  |  |
| Giavedoni et al  2012  UK | COPD patients | NMES (11)  **Within subject comparison**  **Age**: 72.2 ± 3.1 | 30 min/ session  Once/day  Daily  14 days | **Frequency:** 50  **Pulse width:** 400 µs  **Intensity:** maximum tolerated  **Duty cycle:** 8/20s  **Waveform:** biphasic asymmetrical | Quadriceps and vastus medialis | **Primary:**   - Muscle strength (change in maximal voluntary contraction)   **Secondary:**  None | Yes (non-commercial funder) |
|  | **Setting**: Hospital/Patient home | Control (no treatment) (11)  **Within subject comparison**  **Age**: 72.2 ± 3.1  Ethnicity: not reported |  |  |  |  |  |
| Gruther et al  2010  Austria | Critically ill patients | NMES:  **Acute** **group** **AG** (8)  **Long** **term** **group** **LTG** (8)  **Age**:  **AG** (52 ± 10)  **LGT** (61 ± 10) | 30 min/session (during week 0-1)  Then 60 min/ session (during week 2-4)  Once/day  5 days/week  4 weeks | **Frequency:** 50 Hz  **Pulse width:** 350 µs  **Intensity:** maximum tolerated  **Duty cycle:** 8/24s  **Waveform:** biphasic symmetric | Quadriceps of both legs | **Primary:**   - Muscle layer thickness   **Secondary:**  None | No |
|  | **Setting**: ICU | Control (Sham): **AG** (9)  **LGT** (8)  **Age**:  **AG** (48 ± 12)  **LGT** (64 ± 8)  Ethnicity: not reported |  |  |  |  |  |
| Harbo et al 2018  Denmark | Guillain-Barre syndrome | NMES (16)  **Within subject comparison**  **Age:** 52.6 ± 20.4 | 15 min MFS followed by 40 min NMES/day.  Once/day  6-7 days/week  Until discharge | **Frequency:** 3  MFS: 1 Hz  NMES: 10, 40, 60, and 3 HZ  **Pulse width:**  MFS: 250 µs  NMES: 0.3 ms  **Intensity:** highest tolerated level  **Duty cycle:**  MFS: 3/6s  NMES: not reported  **Waveform:**  MFS: not reported  NMES: rectangular dual-phase pulses | Quadriceps | **Primary:**   - Muscle CSA   **Secondary:**   - Muscle strength | No |
|  | **Setting:** Hospital | Control (no treatment)  **Within subject comparison**  **Age:** 52.6 ± 20.4  Ethnicity: not reported |  |  |  |  |  |
| Hardy et al  2022  UK | Patients undergoing abdominal surgery | NMES (15)  **Within subject comparison**  **Age:** 66 ± 5.9 | 15 min/ session  Twice/day  Daily  4 days | **Frequency:** 30 Hz  **Pulse width:** not reported  **Intensity:** visible activity and involuntary knee movement  **Duty cycle:** 1/1s  **Waveform:** not reported | Lateral quadriceps | **Primary:**   - Muscle CSA - Muscle thickness   **Secondary:**   - Muscle architecture measures (muscle thickness, pennation angle and fascicle length). - Muscle strength - Physical activity level - Patient feedback | Yes (non-commercial funder) |
|  | **Setting:** Hospital | Control (no treatment)  **Within subject comparison**  **Age:** 66 ± 5.9  Ethnicity: not reported |  |  |  |  |  |
| Kho et al  2015  USA | Critically ill patients on mechanical ventilation | NMES + UR (16)  **Age**: 54 ± 16 | 60 min/day (one session OR two-30 min sessions)  Daily  Until ICU discharge  30 min/session  Once/day  Daily  8 days (3 preoperative and 5 postoperative) | **Frequency:** 50 Hz  **Pulse width:** 400 µs (quadriceps) and 250 µs (tibialis anterior and gastrocnemius)  **Intensity:** visible contraction or gradually increased to reach maximum value  **Duty cycle:** 5/10s (quadriceps) and 5/5s (tibialis anterior and gastrocnemius)  **Waveform:** biphasic asymmetric, rectangular waveform | Quadriceps, tibialis anterior and gastrocnemius of both legs | **Primary:**   - Muscle strength   **Secondary:**   - Dynamometry muscle strength - Hand grip - Functional status score for ICU (FSS-ICU) - Maximum walking distance (1000 ft) - ICU and hospital length of stay. | Yes (both non-commercial and commercial funders) |
|  | **Setting**: ICU | Control (UR) (18)  **Age**: 56 ± 18  Ethnicity (total): 20 (58%) were white. |  |  |  |  |  |
| Kitamura et al  2019  Japan | Patients after cardiovascular surgery | NMES (60)  **Age , median (IQR):** 67 (55-74) | 30 min/session  Once/day  Daily  8 days (3 preoperative and 5 postoperative | **Frequency:** not reported  A direct electrical current with asymmetric and biphasic square waveform was delivered for 0.4 seconds followed by a 0.6-second pause. Ten pulse trains (10 seconds) were delivered to each muscle with 30-secondintervals and repeated for 30 minutes of a session.  **Intensity:**10-20% of MVC  **Waveform:** biphasic symmetric, square waveform | Quadriceps and triceps surae of both legs | **Primary:**   - Knee extensor isometric strength. - Concentration of 3-methylhistidine corrected for urinary creatinine (3-MH/Cre)   **Secondary:**   - 10-minute walking speed - Grip strength | Yes (non-commercial funder) |
|  | **Setting**: Hospital | Control (UR) (59)  **Age , median (IQR):** 70 (61-77)  Ethnicity: not reported |  |  |  |  |  |
| Nakanishi et al  2020  Japan | Critically ill patients on mechanical ventilation | NMES + mobilisation (17)  **Age**: 73 ± 3 | 30 min/session  Once/day  Daily  5 days | **Frequency:** 20 Hz (200Hz at the start of energization)  **Pulse width:** 650 µs  **Intensity:** visible contraction  **Duty cycle:** 0.4/0.6s  **Waveform:** square wave on the positive side and exponential decay wave on the negative side | Biceps brachii and rectus femoris- bilaterally | **Primary:**   - Muscle mass.   **Secondary:**   - Muscle strength and ICU-AW occurrence - ICU mobility - Length of hospitalization - Amino acid. | Yes (non-commercial funder) |
|  | **Setting**: ICU | Control (mobilisation) (19)  **Age**: 66 ± 3  Ethnicity: not reported |  |  |  |  |  |
| Poltavskaya et al  2022  Russia | Heart failure patients | NMES (22)  **Age**: 64.50 ± 11 | 30, 40, 60, 90 min/session  Once/day  5 days/week  Until discharge | **Frequency:** 25 ± 1 Hz  **Pulse width:** 1 ±0.05 ms  **Intensity:** visible contraction and increased gradually to attain maximum tolerable contraction  **Duty cycle:** 1±0.1/2±0.1s  **Waveform:** bipolar symmetrical rectangular electrical impulses | Femoral and tibial muscles of both legs | **Primary:**   - 6-minute walking test - QoL   **Secondary:**   - Safety and adverse events | Yes (non-commercial funder) |
|  | **Setting:** Hospital | Control (Sham) (23)  **Age**: 68.9 ± 9  Ethnicity: not reported |  |  |  |  |  |
| Poulsen et al  2011  Denmark | Septic shock patients | NMES (8)  **Within subject comparison**  Age, median IQR :67 (64-72) | 60 min/session  Once/day  Daily  7 days | **Frequency:** 35 Hz  **Pulse width:** 300 µs  **Intensity:** visible muscle contraction.  **Duty cycle:** 4/6s  **Waveform:** biphasic pulses. | Quadriceps- unilaterally | **Primary:**   - Muscle volume   **Secondary:**  None | Yes (both non-commercial and commercial funders) |
|  | **Setting**: ICU | Control (ICU care) (8)  **Within subject comparison**  Age, median IQR :67 (64-72)  Ethnicity: not reported |  |  |  |  |  |
| Rodriguez et al  2012  Argentina | Septic patients requiring mechanical ventilation | NMES (14)  **Within subject comparison**  **Age, median IQR**: 72 (63-80) | 30min/session  Twice/day  Daily  Until successful extubation | **Frequency:** 100 Hz  **Pulse width:** 300 µs  **Intensity:** gradually increased until visible muscle contraction, pain threshold, or maximum value  **Duty cycle:** 2/4s  **Waveform:** biphasic waves | Brachial biceps and vastus medialis- unilaterally | **Primary:**   - Arm and leg circumferences - Biceps thickness - Muscle strength   **Secondary:**  None | Yes (non-commercial funder) |
|  | **Setting**: ICU | Control (no treatment) (14)  **Within subject comparison**  **Age, median IQR**: 72 (63-80)  Ethnicity: not reported |  |  |  |  |  |
| Routsi et al  2010  Greece | Critically ill patients | NMES (24)  **Age**: 55 ± 20 | 55 min (5 min warm up & recovery)/session  Once/day  Daily  Until ICU discharge | **Frequency:** 45 Hz  **Pulse width:** 400 µs  **Intensity:** visible or palpable contraction  **Duty cycle:** 12/6s  **Waveform:** Biphasic symmetric | Vastus medialis, lateralis and peroneous longus of both legs | **Primary:**   - Muscle strength   **Secondary:**  None | Yes (non-commercial funder) |
|  | **Setting**: ICU | Control (no treatment) (28)  **Age**: 59 ± 21  Ethnicity: not reported |  |  |  |  |  |
| Segers et al  2021  Belgium | Critically ill patients | NMES + UR (47)  **Within subject comparison**  **Age**: 60 ± 15 | 1 h (5 min warm up & recovery)/ session  Once/day  Daily  7 days | **Frequency:** 45 Hz (4 Hz warm up, recovery and rest phase).  **Pulse width:** 350 µs  **Intensity:** maximum tolerated  **Duty cycle:** 8.5/12s  **Waveform:** not reported | Quadriceps- unilaterally | **Primary:**   - Muscle mass.   **Secondary:**   - Muscle strength - Morphological and molecular markers (histological, gene and protein expressions. | No |
|  | **Setting**: ICU | Control (UR) (47)  **Within subject comparison**  **Age**: 60 ± 15  Ethnicity: not reported |  |  |  |  |  |
| Silva et al  2019  Brazil | Traumatic brain injury patients on mechanical ventilation | NMES + PT (30)  **Age, mean (95% CI)**: 30 (27 to 33) | 25 min/session  Once/day  Daily  14 days | **Frequency:** 100 Hz  **Pulse width:** 400 µs  **Intensity:** maximum tolerated to get muscle response  **Duty cycle:** 5/25s  **Waveform:** biphasic | Quadriceps, hamstring, tibialis anterior, and gastrocnemius muscles of both legs | **Primary:**   - Muscle architecture (muscle thickness and echogenicity ) - The presence of Neuromuscular electrophysiological disorder - Evoked peak force   **Secondary:**   - Plasma level of systematic inflammation. - Catabolic responses. - Mechanical ventilation time - Length of stay in ICU and hospital. - ICU mortality rate | Yes (non-commercial funder) |
|  | **Setting**: ICU | Control (PT) (30)  **Age, mean (95% CI)**: 33 (29 to 37)  Ethnicity: not reported |  |  |  |  |  |
| Strasser et al  2009  Austria | Patients who underwent abdominal surgery | NMES (18)  **Within subject comparison**  **Age**: 60 ± 10 | 30 min/session  Once/day  Daily  4 days | **Frequency:** 50 Hz  **Pulse width:** 250 µs  **Intensity:** maximum tolerated  **Duty cycle:** 8/4s  **Waveform:** not reported | Quadriceps- unilaterally. | **Primary:**   - The mRNA level of IGF-1Ea   **Secondary:**   - The mRNA level of MGF, total RNA content, total protein content, ubiquitin-conjugated proteins, proteasome activity | Yes (non-commercial funder) |
|  | **Setting**: Hospital | Control (Sham) (18)  **Within subject comparison**  **Age**: 60 ± 10  Ethnicity: not reported |  |  |  |  |  |
| Suetta et al 2004, 2008,  2010  Denmark | Subjects were scheduled for unilateral hip replacement operation. | NMES + UR (10)  **Age, mean (range)**: 69 (60-75) | 60 min/day  Once/day  Daily  12 weeks | **Frequency:** 40 Hz  **Pulse width:** 250 µs  **Intensity:** Maximum tolerance  **Duty cycle:** 10/20s  **Waveform:** biphasic current | Quadriceps of the operated side | **Primary:**   - CSA - Muscle thickness - Muscle strength - Muscle force development - Length of hospitalisation - Functional performance (walking test, stair climbing, sit-to-stand) - IGF-I   **Secondary:**  None | Yes (non-commercial funder) |
|  | **Setting:** Hospital/ Patient home | Control (UR) (9)  **Age, mean (range)**: 69 (62-78)  Ethnicity: not reported |  |  |  |  |  |
| Sumin et al  2020  Russia | Patients with postoperative complications after cardiovascular surgery | NMES + UR (18)  **Age, median (IQR)**: 61.5 (52-71) | 90 min at least (5 min warm up)/session  Once/day  Daily  Until hospital discharge | **Frequency:** 45 Hz  **Pulse width:** not reported  **Intensity:** visible or palpable contraction  **Duty cycle:** 12/5s  **Waveform:** rectangular pulses | Quadriceps of both legs | **Primary:**   - Isometric knee extensors strength.   **Secondary:**   - Handgrip strength - Knee flexors strength - CSA - 6-minute walking distance | Yes (non-commercial funder) |
|  | **Setting**: ICU/Hospital ward | Control (UR) (19)  **Age, median (IQR)**: 64 (60-68)  Ethnicity: not reported |  |  |  |  |  |
| Takino et al  2023  Japan | Patients with diabetes after cardiovascular surgery | NMES (90)  **Age**: 74 ± 5 | 60 min/session  Once/day  Daily  5 days | **Frequency:** 200 followed by 20 Hz  A direct electrical current with asymmetric and biphasic square waveform was delivered for 0.4s, followed by a 0.6s pause. Ten pulse trains (10s) were delivered to each muscle at 30s intervals for 60min  **Intensity:** visible muscle contraction at the maximum tolerated intensity  **Waveform:** symmetric, biphasic square waveform | Quadriceps and triceps surae of both legs | **Primary:**   - Isometric knee extensors strength.   **Secondary:**   - 10-minute walking speed - Grip strength | Yes (no funding received) |
|  | **Setting:** Hospital | Control (Sham) (90)  **Age**: 74 ± 5  Ethnicity: not reported |  |  |  |  |  |
| Tanaka et al  2022  Japan | Frail old patients with acute decompensated heart failure | NMES + EM (15)  **Age**: 82.5 ± 4 | 30-40 min/session  Once/day  5 days/week  Until discharge (up to 2 weeks) | **Frequency:** 20  **Pulse width:** 250 µs  **Intensity:** Visible contraction  **Duty cycle:** 5/2s  **Waveform:** not reported | Quadriceps, hamstrings, tibialis anterior, and triceps surae muscles of both legs (all lower extremity muscles) | **Primary:**   - Change in muscle strength.   **Secondary:**   - Gait speed - 6-minute walking distance - Handgrip strength - Cognitive function - Clinical and adverse events | Yes (non-commercial funder) |
|  | **Setting**: Hospital | Control (EM) (16)  **Age**: 83.3 ± 5.5  Ethnicity: not reported |  |  |  |  |  |
| Vivodtzev et al  2006  France | COPD patients | NMES + UR (9)  **Age**: 59 ± 15 | >30 min (5 min warm up)/session  Once/day  4 days/week  4 weeks | **Frequency:** 35 Hz (5 Hz warm up and rest periods)  **Pulse width:** 400 µs  **Intensity:** maximum tolerated  **Duty cycle:** 7/8s  **Waveform:** biphasic symmetric, square pulsed current | Quadriceps of both legs | **Primary:**   - Pulmonary function tests ( FEV_1_, FVC) - QoL - Muscle strength - Muscle mass - 6-minute Walking distance   **Secondary:**  None | Yes (non-commercial funder) |
|  | **Setting**: Hospital | Control (UR) (8)  **Age**: 68 ± 12  Ethnicity: not reported |  |  |  |  |  |
| Zulbaran-Rojas et al  2022  USA | Covid-19 patients | NMES (8)  **Age**: 66.75 ± 9.81 | 60 min/session  Once/day  Daily  14 days (NMES was only for 9 days) | **Frequency:** 20-121 Hz  **Pulse width:** 400-1400 µs  **Intensity:** 50 V  **Duty cycle:** not reported  **Waveform:** high voltage pulsed alternative current (HVPAC) in the shape of an asymmetrical damped sinusoidal biphasic pulsed waveform | Gastrocnemius of both legs | **Primary:**   - Muscle endurance - Ankle strength - Risk of fall - Oxygen saturation - Safety and feasibility outcomes.   **Secondary:**  None | Yes (commercial funder) |
|  | **Setting:** ICU | Control (Sham) (8)  **Age**: 62.88 ± 9.51  Ethnicity (total): 9 (56%) were Hispanic, 5 (31%) African American, 1 (6%) Caucasian, and 1 (6%) Asian. |  |  |  |  |  |

Abbreviation: COPD, chronic obstructive pulmonary disease; ICU, intensive care unit; IQR, interquartile range; CSA, cross-sectional area; PT, physiotherapy; EM, early mobilisation; ROM, range of motion; MFS, direct muscle fibre stimulation; UR, usual rehabilitation, ICU-AW, intensive care unit acquired weakness; CI, confidence interval; IGF-I, insulin-like growth factor-I; IGF-1Ea, insulin-like growth factor-1EA, MGF, mechano growth factor; FEV_1_, forced expiratory volume in one second; FVC, forced vital capacity; MHC, myosin heavy chain; CRP, C- reactive protein; IL, interleukin; TNF, tumor necrosis factor; TUG, timed up and go; Qol, quality of life; FOXO1, forkhead box protein O1; MAFbx, muscle atrophy F-box; MuRF1, muscle RING-fingerprotein-1; mTOR, mammalian target of rapamycin; P70S6K, p70S6 kinase.

## Funnel plot of muscle strength


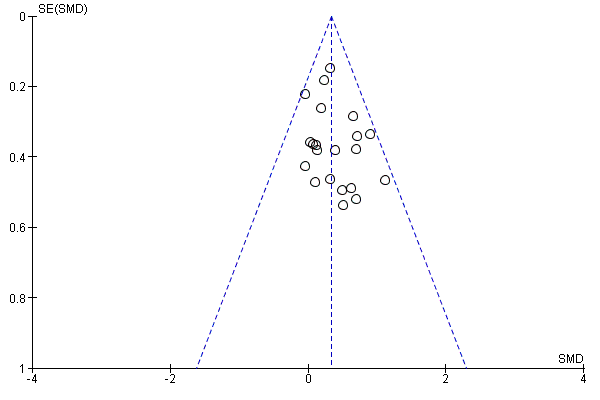
Figure S1. Funnel plot of muscle strength

## Subgroup analyses

Muscle strength

In subgroup analysis, both ICU and non-ICU studies showed a significant benefit of NMES over control. For ICU studies there was a small effect size (SMD 0.31; 95% CI [0.09, 0.52]; p<0.01) with no heterogeneity (I^2^= 6%). For non-ICU studies, there was also a small effect size (SMD 0.30; 95% CI [0.10, 0.50]; p <0.005) with no heterogeneity (I^2^= 0%).


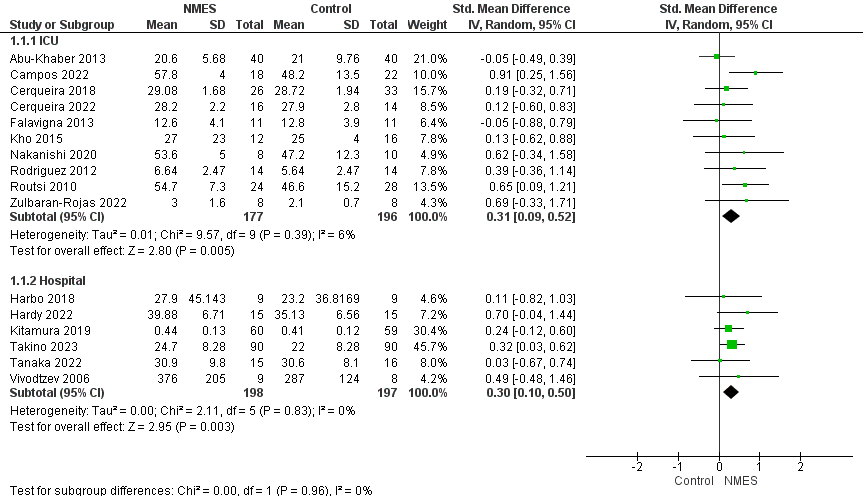
Figure S2. Forest plot: Muscle strength (ICU vs non-ICU subgroup).

Muscle mass

In subgroup analysis, both ICU and non-ICU studies showed a significant benefit of NMES over control. For ICU studies there was a moderate effect size (SMD 0.62; 95% CI [0.21, 1.04]; p<0.005) with moderate heterogeneity (I^2^= 66%). For non-ICU studies, there was a large effect size (SMD 1.56; 95% CI [0.05, 3.06]; p<0.05) with high heterogeneity (I^2^= 84%).


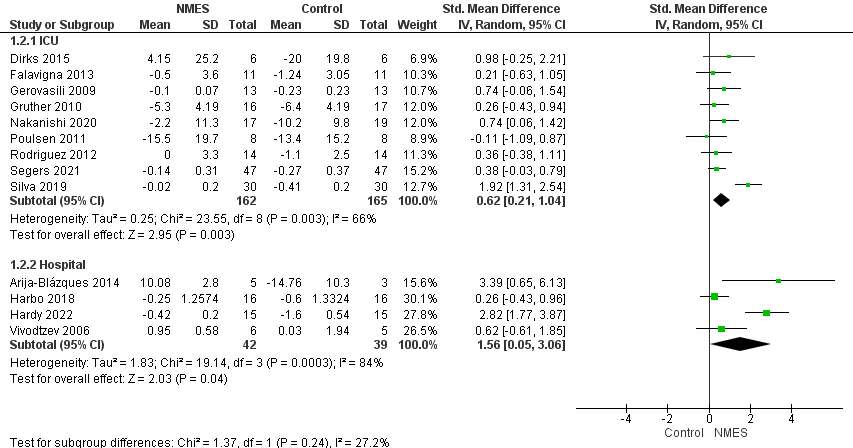
Figure S3. Forest plot: Muscle mass (ICU vs non-ICU subgroup).

Walking performance

In subgroup analysis, ICU studies showed a non-significant difference between groups with no heterogeneity (I^2^= 0%) and no / negligible effect (SMD 0.06; 95% CI [-0.31, 0.43]; p>0.05), but in non-ICU settings there was a significant difference with moderate heterogeneity (I^2^= 61%) and a small effect size (SMD 0.49; 95% CI [0.15, 0.83]; p<0.01).


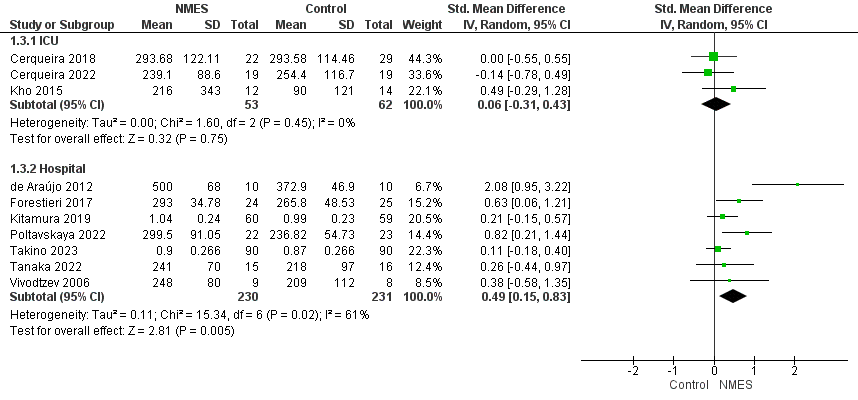
Figure S4. Forest plot: Walking performance (ICU vs non-ICU subgroup).

## Sensitivity analyses

Muscle Strength

A similar meta-analysis result was obtained in a sensitivity analysis excluding two studies reporting a change score instead of post intervention score and five studies for which parametric statistics were calculated from 95%CI, p-value, and median and ranges (SMD 0.24; 95% CI [0.07, 0.41]; P < 0.01, I^2^= 0%, Figure S4.1).


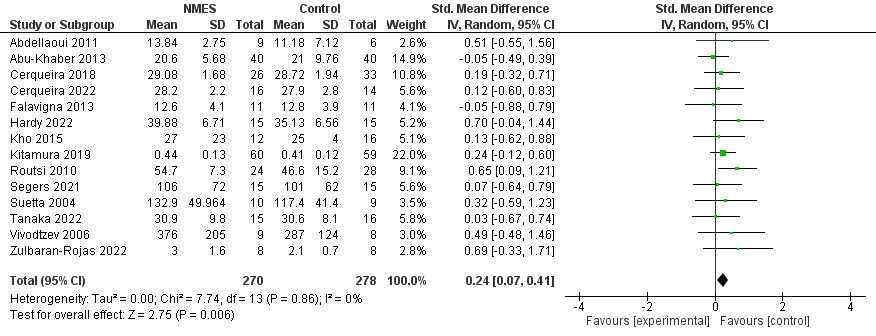


Figure S5. Forest plot: Muscle strength (sensitivity analysis).

Muscle Mass

A significant finding was obtained in a sensitivity analysis excluding two studies reporting post intervention score instead of a change score and two studies using data approximated from non-parametric statistics (SMD 0.93; 95% CI [0.43, 1.42]; P < 0.0005, I^2^= 76%, Figure S4.2).


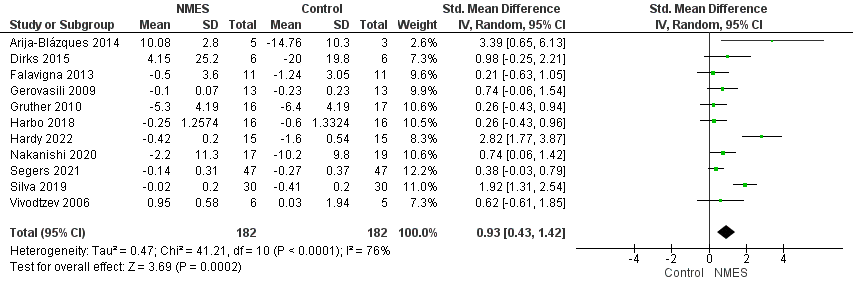
Figure S6. Forest plot: Muscle mass (sensitivity analysis).

Function

*Walking performance*

A significant finding was obtained in a sensitivity analysis excluding three studies using data approximated from non-parametric statistics (SMD 0.43; 95% CI [0.17, 0.70]; P < 0.005, I^2^= 39%, Figure S4.3).


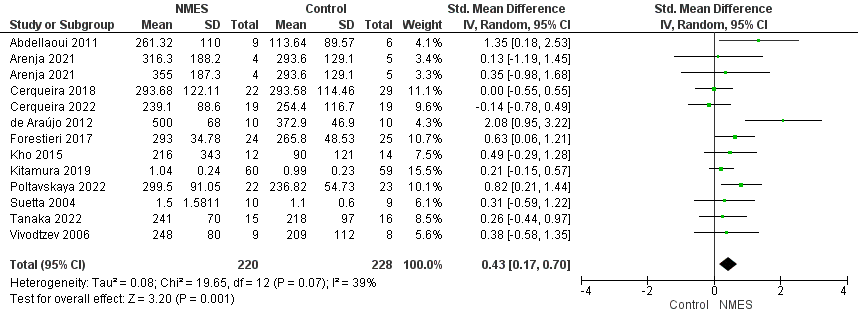
Figure S7. Forest plot: Walking performance (sensitivity analysis).

*Functional mobility*

A non-significant finding was obtained in a sensitivity analysis excluding five studies using data approximated from non-parametric statistics (SMD 0.22; 95% CI [-0.15, 0.59]; P > 0.05, I^2^= 55%, Figure S4.4).


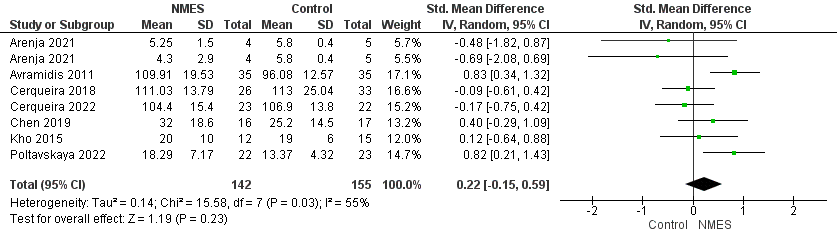
Figure S8. Forest plot: Functional mobility (sensitivity analysis)
